# Transcriptomic Analysis of Human Retinal Detachment Reveals Both Inflammatory Response and Photoreceptor Death

**DOI:** 10.1371/journal.pone.0028791

**Published:** 2011-12-09

**Authors:** Marie-Noëlle Delyfer, Wolfgang Raffelsberger, David Mercier, Jean-François Korobelnik, Alain Gaudric, David G. Charteris, Ramin Tadayoni, Florence Metge, Georges Caputo, Pierre-Olivier Barale, Raymond Ripp, Jean-Denis Muller, Olivier Poch, José-Alain Sahel, Thierry Léveillard

**Affiliations:** 1 INSERM, U968, Paris, France; 2 UPMC Univ Paris 06, UMR_S 968, Institut de la Vision, Paris, France; 3 CNRS, UMR_7210, Paris, France; 4 Unité Rétine, Uvéite et Neuro-Ophtalmologie, Département d'Ophtalmologie, Centre Hospitalier Universitaire de Bordeaux, Bordeaux, France; 5 Laboratoire de BioInformatique et Génomique Intégratives, CNRS UMR_7104, Institut de Génétique et de Biologie Moléculaire et Cellulaire, Strasbourg, France; 6 CEA, LIST, Information, Models and Learning Laboratory, Gif-sur-Yvette, France; 7 Département d'Ophtalmologie, Hôpital Lariboisière, Paris, France; 8 Vitreo-retinal Unit, Moorfields Eye Hospital, London, United Kingdom; 9 Fondation Ophtalmologique Adolphe de Rothschild, Paris, France; 10 Centre Ophtalmologique des Quinze-Vingts, Paris, France; Dalhousie University, Canada

## Abstract

**Background:**

Retinal detachment often leads to a severe and permanent loss of vision and its therapeutic management remains to this day exclusively surgical. We have used surgical specimens to perform a differential analysis of the transcriptome of human retinal tissues following detachment in order to identify new potential pharmacological targets that could be used in combination with surgery to further improve final outcome.

**Methodology/Principal Findings:**

Statistical analysis reveals major involvement of the immune response in the disease. Interestingly, using a novel approach relying on coordinated expression, the interindividual variation was monitored to unravel a second crucial aspect of the pathological process: the death of photoreceptor cells. Within the genes identified, the expression of the major histocompatibility complex I gene *HLA-C* enables diagnosis of the disease, while *PKD2L1* and *SLCO4A1* -which are both down-regulated- act synergistically to provide an estimate of the duration of the retinal detachment process. Our analysis thus reveals the two complementary cellular and molecular aspects linked to retinal detachment: an immune response and the degeneration of photoreceptor cells. We also reveal that the human specimens have a higher clinical value as compared to artificial models that point to IL6 and oxidative stress, not implicated in the surgical specimens studied here.

**Conclusions/Significance:**

This systematic analysis confirmed the occurrence of both neurodegeneration and inflammation during retinal detachment, and further identifies precisely the modification of expression of the different genes implicated in these two phenomena. Our data henceforth give a new insight into the disease process and provide a rationale for therapeutic strategies aimed at limiting inflammation and photoreceptor damage associated with retinal detachment and, in turn, improving visual prognosis after retinal surgery.

## Introduction

Retinal detachment (RD) is a potentially blinding condition characterized by the subretinal accumulation of fluid in a space created between the neurosensory retina at the level of photoreceptor cells and the underlying retinal pigment epithelium (RPE). In most cases, RD occurs secondary to a full thickness retinal break and is hence called “rhegmatogenous” (from the Greek word rhegma, a “rent”). The incidence of RD is strongly correlated with age, myopia and vitreoretinal degenerations. Annual incidence is estimated at 10.5/100,000 [1] and the treatment of rhegmatogenous RD remains to this day exclusively surgical. However, despite retinal reattachment after surgery, visual outcome remains below expectation in many cases and patients often report permanent alterations in colour perception and/or severe loss of visual acuity due to the loss of photoreceptor cells [2], [3], [4], [5]. The physical separation of photoreceptors from RPE cells indeed results in the interuption (disruption) of the transfer of nutrients to photoreceptors, thereby inducing chronic disturbances in cellular metabolism. Over a period of few days, retinal remodeling occurs, photoreceptor outer segments shorten and progressive death through apoptosis takes place [6], [7], [8]. The use of adjuvant neuroprotective molecules that would limit the damage to photoreceptors in combination with surgery has hence been proposed [9]. Besides the loss of photoreceptors secondary to the detachment itself, an inflammatory response develops during RD that leads to Proliferative Vitreo-Retinopathy (PVR), a clinical outcome resulting from the formation of contractile cellular membranes on both surfaces of the retina and in the vitreous. PVR in turn accelerates photoreceptor degeneration and may even cause failure of the retinal reattachment after surgery [2], [10]. Pilot studies aimed at preventing PVR with anti-inflammatory agents have been conducted, but with only limited success [11], [12], [13].

The main therapeutic challenge in RD is to limit photoreceptor cell damage and PVR occurrence (or recurrence). We wished to identify some of the most appropriate molecules that could be used efficiently in combination with surgery and improve final visual outcome. We used here a differential transcriptomic analysis to identify target genes with modified expression following RD. Human retinal specimens were collected from patients undergoing retinal surgery for severe retinal detachment with PVR and requiring retinectomy using a protocol designed for this study, whereas normal control retina specimens were obtained from post-mortem donors. We validated these specimens by measuring via quantitative RT-PCR the expression of genes known to be modified by RD. We then performed a global analysis with data obtained from 19 RD RNA preparations and 19 controls that were hybridized to Affymetrix genechip arrays. A differential transcriptomic analysis was used to identify target genes with modified expression following RD. The data were first treated to highlight genes with statistical difference between the two groups (RD and controls) using the false discovery rate (FDR) method. As a complementary approach, we used the inherent variability between individuals as information for identifying co-regulated genes using a method based on mutual information (MI) [14]. Interestingly, we found that using the same set of data, the FDR method points to the involvement of an inflammatory process linked to PVR, while the MI method identifies more specifically genes with a decrease in expression linked to photoreceptor loss. These data point to novel therapeutic strategies. We further show that these biomarkers can be used to evaluate the duration of the retinal detachment process.

## Results

### Validation of the human retinal detachment specimens by quantitative RT-PCR

**Figure 1:**
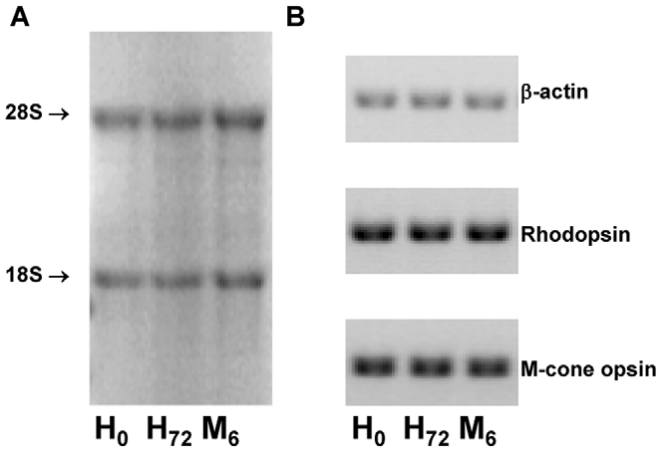
The Kit "jouRNAl" for RNA conservation. (A) Denaturing agarose gel electrophoresis of RNAs purified from porcine retinas. Retinas were immerged in 2.4 ml of 6 M GHCl and purified: i) immediately after immersion (H_0_) or ii) after storage at room temperature during 72 hours (H_72_) or iii) after storage at room temperature during 72 hours and then at -80°C during 6 months (M_6_). 28 S and 18 S ribosomal RNA species have a ratio of 2 indicating the absence of RNA degradation. (B). Agarose gel electrophoresis of RT-PCR amplification products (β-actin, Rhodopsin, M-cone opsin). Similar products were obtained for the 3 samples.

Retinal samples resulting from retinectomy are not used for any clinical analysis and are considered *res nullius* and currently discarded (Table 1). We have used this biological material to perform a transcriptomic analysis of RD. In order to ease the recovery of RNA from the surgical specimens, we developed "jouRNAl" a method that allows RNA conservation between the surgical blocks and the laboratory. To test the method, we immersed 1 cm^2^ of porcine retinas in 2.4 ml of 6 M Guanidium chloride (GHCl) [15], homogenized the specimens and stored them in two different conditions: i) at room temperature for 72 hours, and ii) at room temperature during 72 hours followed by storage at −80°C for 6 months. We found that no RNA degradation had occurred in any of these conditions (Figure 1A). Quantitative RT-PCR demonstrated no modifications in the amount of rhodopsin or M-opsin mRNAs during storage (Figure 1B). The clinical value of the human RD specimens was assessed by quantitative RT-PCR. Transcripts encoding photoreceptor markers were drastically down-regulated in RD samples. Expression of the four visual pigments (i.e. rhodopsin, S-, M- and L-cone opsins) is decreased in RD patients by 25, 8, 9 and 8-fold, respectively as is the rod-specific transducin (Table 2). *NXNL1, *the gene encoding the trophic factor Rod-derived Cone Viability factor (RdCVF) [16], [17] is down-regulated 3-fold. Neuronal markers such as cytochrome oxidase or synaptophysin are also down-regulated as reported [18] **.** Apoptosis during RD and PVR was documented [6], [7]. Apoptosis signal-regulating kinase 1 (ASK1), a mitogen-activated protein kinase that plays a role in oxidative stress [19], is up-regulated 1.9-fold in the RD group (Table 2). Muller Glial Cells (MGCs) have been shown to proliferate and become hypertrophic during RD expressing high level of Glial Fibrillary Acidic Protein (GFAP) [18], [20], [21], as confirmed in the specimens analyzed here (7.9-fold increase). Impaired function of MGCs is correlated with a decrease of glutamine synthetase (GS) as seen here. During RD, RPE cells are activated, migrate, dedifferentiate and proliferate at the surface of the detached retina, exerting contractile forces leading to PVR [22], [23], [24]. Hence, whereas RPE cells are not present within the neural retina in controls, RPE markers are found in the detached retinas. Markers of migrating dedifferentiated RPE cells, *CD68* and the α-smooth actin were increased in RD specimens as reported [25], [26]. PVR associated with severe RD involves both inflammatory and immune responses [27], [28]. The expression of *CD4* and *ICAM1* is increased 2-fold and 27-fold respectively [28], [29], [30], [31]. Matrix MetalloProteinases (MMPs) and their inhibitors (TIMPs) that take part in the remodeling of the extra-cellular matrix during PVR [32], [33], [34], [35] are up-regulated in RD specimens here. Altogether, our quantitative RT-PCR analysis of genes previously reported to be involved in RD validates the clinical quality of the specimens.

**Table 1:** Clinical characteristics of patients with retinal detachment (RD).

Age	Sex	Prior RD surgical procedures	Total RD duration	PVR at surgery
70	M	1 (Vitrectomy + silicone oil tamponade)	1 week	CA2
39	F	1 (Vitrectomy + gas)	3 weeks	CP2–CA2
55	F	1 (Vitrectomy + gas)	1 month	CP4–CA3
73	F	1 (Vitrectomy + gas)	1 month	CA2
36	M	1 (Vitrectomy + silicone oil tamponade)	1 month	CP2–CA2
66	M	No	2 months	CP12–CA10
54	M	2 [(Cryotherapy + gas)/(Vitrectomy + silicone oil tamponade)]	2 months	CP2–CA3
23	M	No	2 months	CP4–CA4
69	M	1 (Vitrectomy + silicone oil tamponade)	3 months	CP2–CA2
65	M	1 (Vitrectomy + silicone oil tamponade)	3 months	CA3
58	M	1 (Vitrectomy + silicone oil tamponade)	3 months	CP2–CA2
55	M	2 [(Cryo + scleral buckling)/Vitrectomy + gas)]	3 months	CP12–CA9
31	M	No	>3 months	CP4
74	F	No	4 months	CP3–CA2
32	M	2 [(Cryo + scleral buckling)/Vitrectomy + gas)]	6 months	CP3–CA3
59	F	2 [(Cryo + scleral buckling)/Vitrectomy + gas)]	6 months	CP3–CA3
53	M	1 (Vitrectomy + gas)	7 months	CP4–CA4
49	M	2 [(Cryo + scleral buckling)/Vitrectomy + gas)]	1 year	CA4
30	M	No	1 year	CP12–CA8

Proliferative vitreoretinopathy (PVR) is graded using the up-dated classification of the Retina Society Terminology Committee by Machemer [22]. There are three grades describing increasing severity of the disease: A, B and C. In patients that all required retinectomy, RDs are always associated with grade C of PVR. Posterior (P) and anterior (A) location of the proliferations are specified. The extent of the abnormalities is detailed by using clock hours. Hence, CP3–CA2 means grade C of PVR with both posterior (P) and anterior (A) abnormalities extended over three (CP3, meaning ¼) and two (CA2) clock hours respectively.

**Table 2:** Comparison of the expression measured by quantitative RT-PCR and by hybridization to array.

Gene Name		Gene Symbol	QRT-PCR			Probeset n°	Affymetrix				
			RD/Ctrs	SE	pvalue		RD/Ctrs		SE		pvalue
**Apoptosis**											
ASK1	*MAP3K5*		1.84	0.09	<0.005	203836_s_at	1.65	0.03		2.5e–5	
**Cellular Stress**											
AlphaB crystallin	*CRYAB*		0.91	0.04	NS	209283_at	1.35	0.03		4.3e–3	
**Glial Cell Response**											
GFAP	*GFAP*		7.96	1.00	<0.0005	203540_at	12.49	0.39		5.4e–10	
GLAST	*SLC1A3*		1.38	0.12	NS	202800_at	2.00	0.04		2.3e–8	
Glutamine synthetase	*GLUL*		0.40	0.02	<0.00005	215001_s_at	0.70	0.01		9.3e–8	
**RPE Cell Response**											
CD68	*CD68*		11.66	1.03	<0.005	NU					
Alpha-smooth muscle actin	*ACTA2*		8.39	0.32	<0.05	200974_at	8.10	0.44		2.5e–4	
**Inflammatory and Immune Mediators**											
CD4	*CD4*		1.82	0.11	<0.05	203547_at	1.72	0.05		1.6e–2	
Intercellular adhesion molecule 1	*ICAM1*		27.24	4.17	<0.0005	202638_s_at	17.10	0.77		3.0e–9	
**Tissue Remodeling**											
Matrix MetalloProteinase 2	*MMP2*		3.86	0.32	<0.0005	201069_at	4.65	0.12		1.0e–9	
Tissue Inhibitor Metalloproteinase 1	*TIMP1*		9.11	0.51	<0.0001	201666_at	9.90	0.23		1.8e–7	
**Cellular Proliferation and Differentiation**											
PCNA	*PCNA*		0.26	0.03	<0.0005	201202_at	0.48	0.01		3.6e–8	
c-fos	*FOS*		0.22	0.04	<0.05	209189_at	0.49	0.04		1.57	
Ornithine decarboxylase	*ODC1*		1.37	0.11	NS	200790_at	1.28	0.02		3.0e–3	
Cyclin A1	*CCNA1*		0.68	0.10	NS	NU					
Cyclin D1	*CCND1*		6.09		<0.01	208712_at	4.00	0.14		1.7e–8	

NS, non statistically significant. NU, Not used since the probesets 203507_at (*CD68*) and 205899_at (*CCNA1*) have relative expression value <10 in any condition.

### Global gene expression analysis indicates an inflammatory process

**Figure 2:**
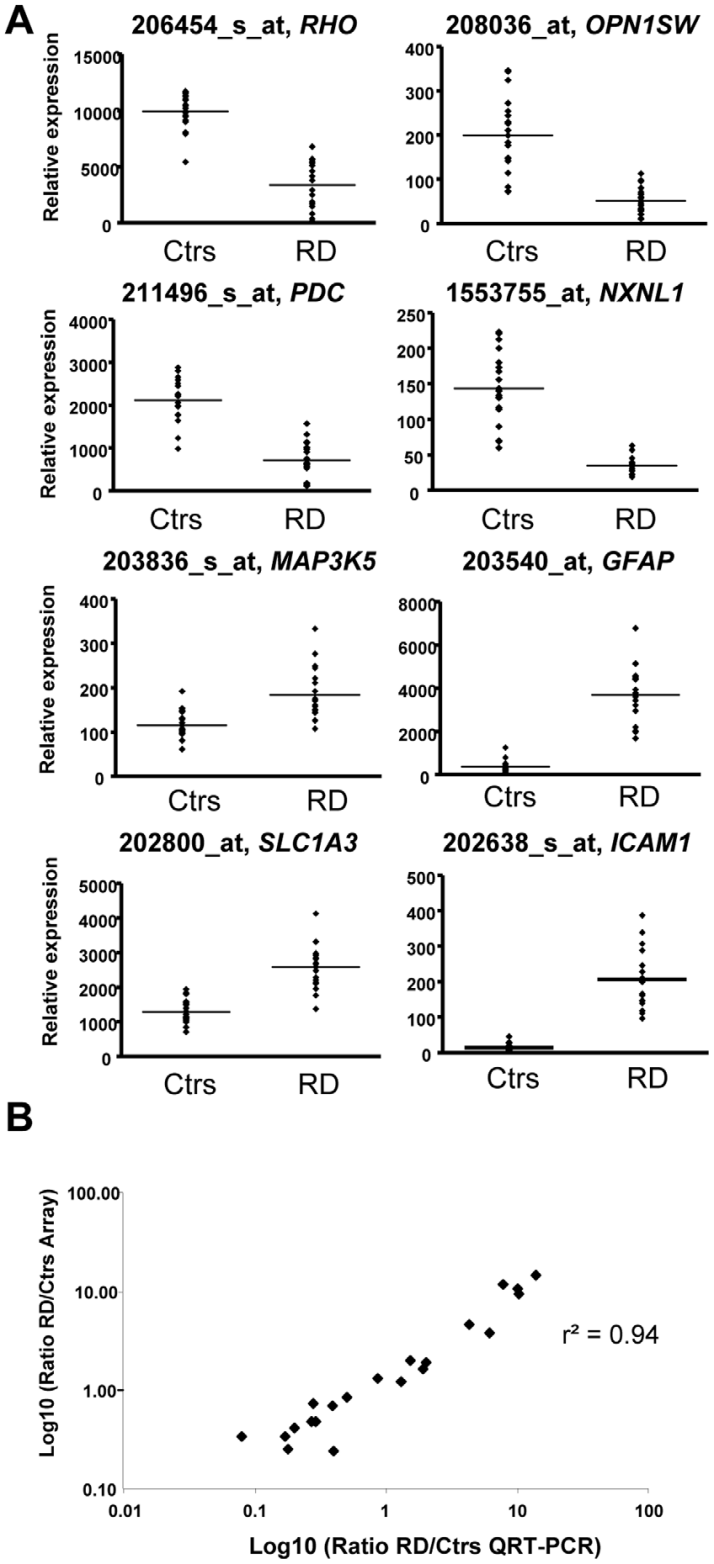
Comparison QRT-PCR/Array results. (A) Expression of Affymetrix probesets for mRNA tested by quantitative RT-PCR. The histograms display controls (Ctrs) and retinal detachment (RD) for each specimen. The horizontal line is the average. (B) Graph displaying the Log10 of ratio retinal detachments versus controls (RD/Ctrs). *CD4* and Cyclin A1 (*CCNA1*) were excluded from the analysis since the values were inferior to 10 in any conditions, while the cone long and middle wave opsin genes (*OPN1LW* and *OPN1MW*) were averaged since the Affymetrix probeset does not distinguish the two genes in tandem on the genome.

After the qualification of the specimens, we hybridized 19 RD and 19 controls RNA preparations to genome array (Affymetrix U133). We then compared the relative expression for probesets corresponding to 21 out of 24 of the genes tested by quantitative RT-PCR. All the examined genes have an expression similar to the quantification made by RT-PCR (Table 2). The photoreceptor markers *RHO*, *OPN1SW*, *PDC* and *NXNL1* were down-regulated, while *GFAP*, GLAST (*SLC1A3*), ASK1 (*MAP3K5*) and *ICAM1* were up-regulated (Figure 2A). The overall results correlated when the Log10 of the RD/Ctrs ratio was plotted for both methodologies (r^2^ = 0.94, Figure 2B). This comparison confirms that the arrays are representative of the RD process.

**Figure 3:**
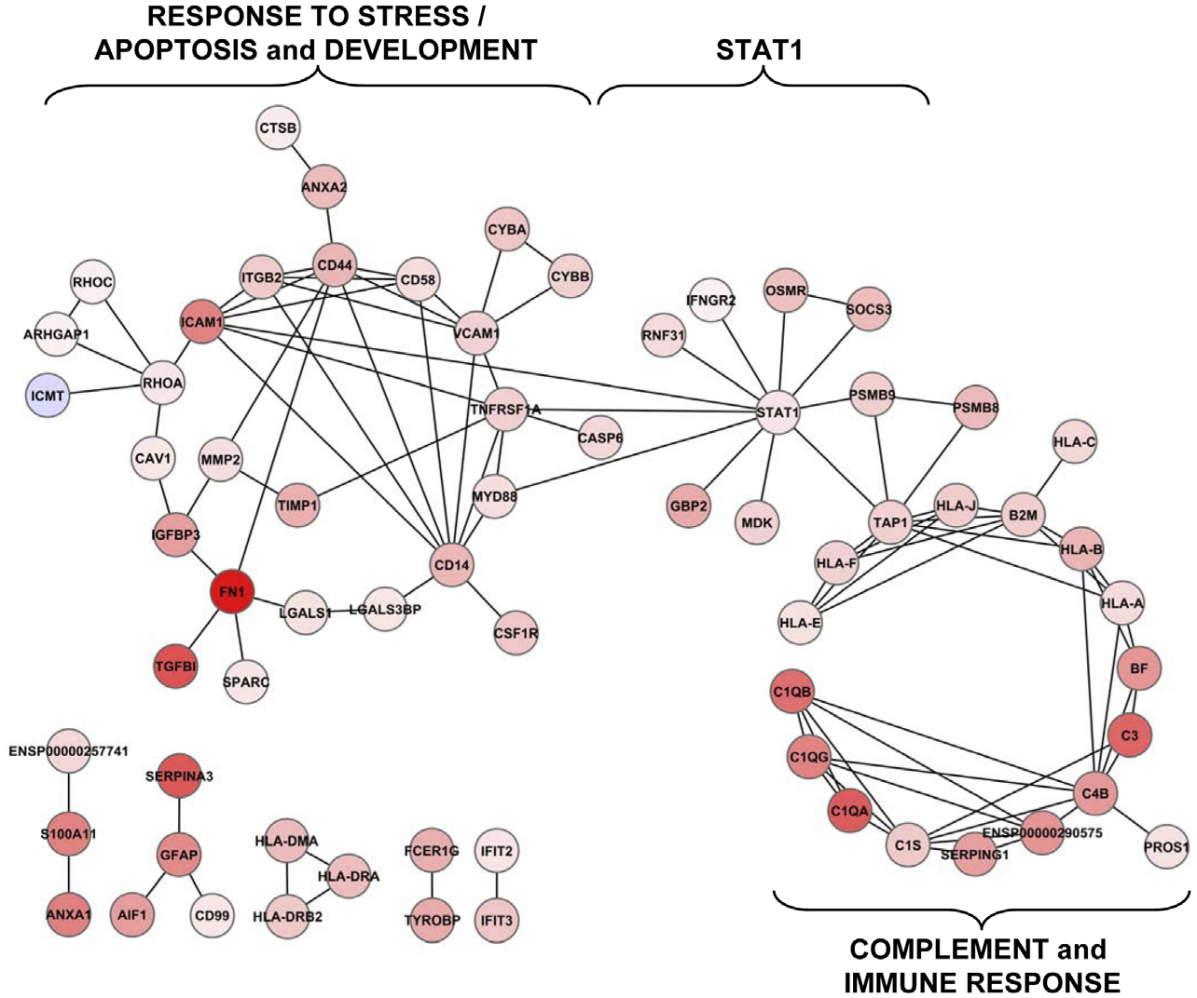
Interactomic analysis of the genes identified by FDR. The interaction between genes identified using the FDR methods. The circles on the left and on the right have been named according to their enrichment in gene ontology terms.

We identified 259 probesets differentially expressed using local FDR [36] (Table S1, fdr259 ps). We observed mostly up-regulated genes by RD (239/259, 92.3%). In order to rule out that this results from the use of post-mortem tissues as controls, we analyzed the interactions between the 259 identified genes in the STRING database [37]. Many of the up-regulated genes were related to an inflammatory process, with the highest fold changes comprising three members of the complement pathway *C1QA*, *C1QB*, *C1QG* and *C3*, five members of the major histocompatibility complex of class II (MHCII) *HLA-A*, *-B*, *-E*, *-F -J*, and the β2-microglobulin (Figure 3). We also tested for enrichment of Gene Ontology (GO) terms and clusters of terms [38]. The two clusters with the highest enrichment scores correspond to immune response and antigen processing (Table 3 and Table S2). The other enriched clusters may translate the same inflammatory process, but the last term (Cell death, 2.02) refers to neuronal degeneration. Thus, the enrichments demonstrate the existence of an immune response following RD and rule out the possibility that the prominence of up-regulated genes results from the difference in tissue processing. In order to evaluate the effect of RD duration on gene expression, we subdivided the RD specimens into early (RD≤1 month), mid-term (1>RD≤3 months) and late (RD>3 months) classes, according to Table 1. Here again, the analysis was dominated by up-regulated genes (Table S2).

**Table 3:** Enrichment scores of clusters of Gene Ontology terms for targeted identified by FDR or MI.

Enrichment Score	Cluster description
**259 targets selected with FDR**	
8.77	Immune response
8.31	Antigen processing
7.23	Response to stress
3.41	Signal transduction
2.55	Carbohydrate binding
2.48	Cell morphogenesis
2.41	Membrane
2.02	Cell death
**266 targets selected with MI**	
4.58	Antigen processing and presentation
2.90	Visual perception
1.83	Organelle outer membrane
1.50	Membrane docking
1.35	Phototransduction

### Co-regulated genes reveal photoreceptor dysfunction and death

**Figure 4:**
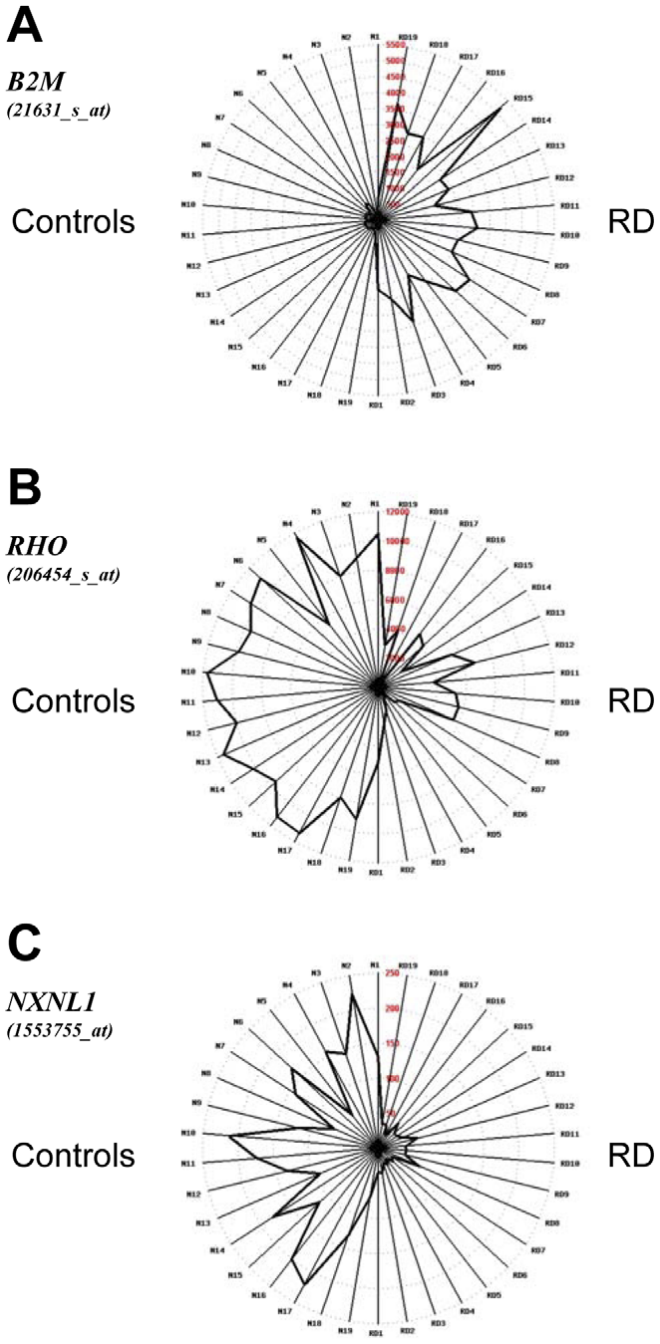
Expression of *B2M*, *RHO* and *NXNL1* in a radar graph display. The graph has been designed using Retinobase. The controls (N1 to N19) and the retinal detached specimens (RD1 to RD19) are displayed on the left and the right of the radars respectively.

The graphic representation of the data using Retinobase [39] shows the all the specimens with RD have an elevated expression of β2-microglobulin (*B2M*), likely corresponding to the degree of inflammatory response (Figure 4A), while rhodopsin (*RHO*) and *NXNL1* are down-regulated. By closely examining the interindividual variability (Figure 4B and C), it seems that the patterns of these two genes somehow matched, in agreement with the fact that *NXNL1* is expressed in a rod-dependent manner [16], [40]. We used the information encoded by the interindividual variability to identify genes that are co-regulated using a novel approach based on mutual information (MI). MI relies on the concept that if two genes are co-regulated, the expression pattern of the first provides information that predicts the expression pattern of the second [14]. When applied to the three stratified classes of RD specimens, early, mid-term and late, this method selected 266 probesets having a normalized MI value above a threshold of 0.5 (Figure S1). Contrary to the FDR analysis, 58.6% of the 266 probesets points to genes that are down-regulated in RD.

### Identification of three biomarkers for retinal detachment

**Figure 5:**
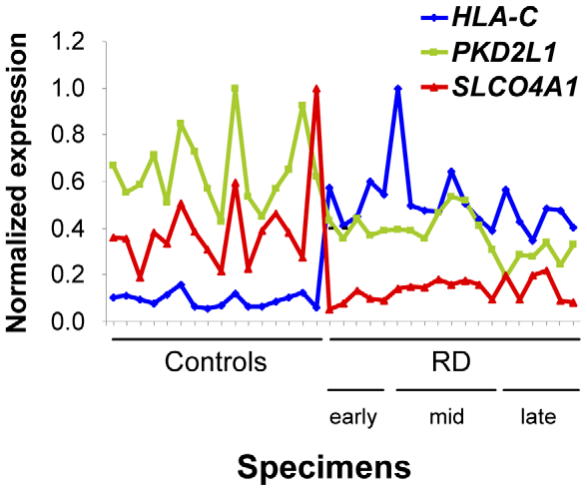
The role of the three identified biomarkers *HLA-C, PKD2L1 and SLCO4A1* as classifiers. Expression of the three RD markers *HLA-C, PKD2L1 and SLCO4A1* in the specimens. The black line represents the classes that are: controls, early, RD≤1 month, mid-term, 1>RD≤3 months and late, RD >3 months.

Since the expression profiles of the 266 probesets are all linked together, we used an iterative calculation to identify the minimal number of probesets that will fully describe the phenomenon (see methods). The three probesets 216526_x_at (*HLA-C*), 221061_at (*PKD2L1*) and 219911_s_at (*SLCO4A1*) are sufficient to describe the whole dataset. These probesets, among which two are down-regulated in RD, score both correlated and anticorrelated genes. We examined the expression of these three probesets with the delay after RD. We noticed that the expression of both *PKD2L1* and *SLCO4A1* is particularly low in the late RD group (Figure 5). Interestingly, while the addition of *PKD2L1* to *HLA-C* reduced the combined normalized MI from 0.603 to 0.596, the inclusion of the third probeset (*SLCO4A1*) resulted in an increase of the normalized MI to 0.611 above the value of *HLA-C* alone. It seems that *PKD2L1* and *SLCO4A1* act in a synergistic manner. This indicates that the up-regulation of *HLA-C* is strictly correlated with RD and that the down-regulation of *SLCO4A1* and *PKD2L1* is informative for the duration of RD with lower expression of *SLCO4A1* at early stage and of *PKD2L1* at the late stage.

### The mutual information analysis points to photoreceptor degeneration

**Figure 6:**
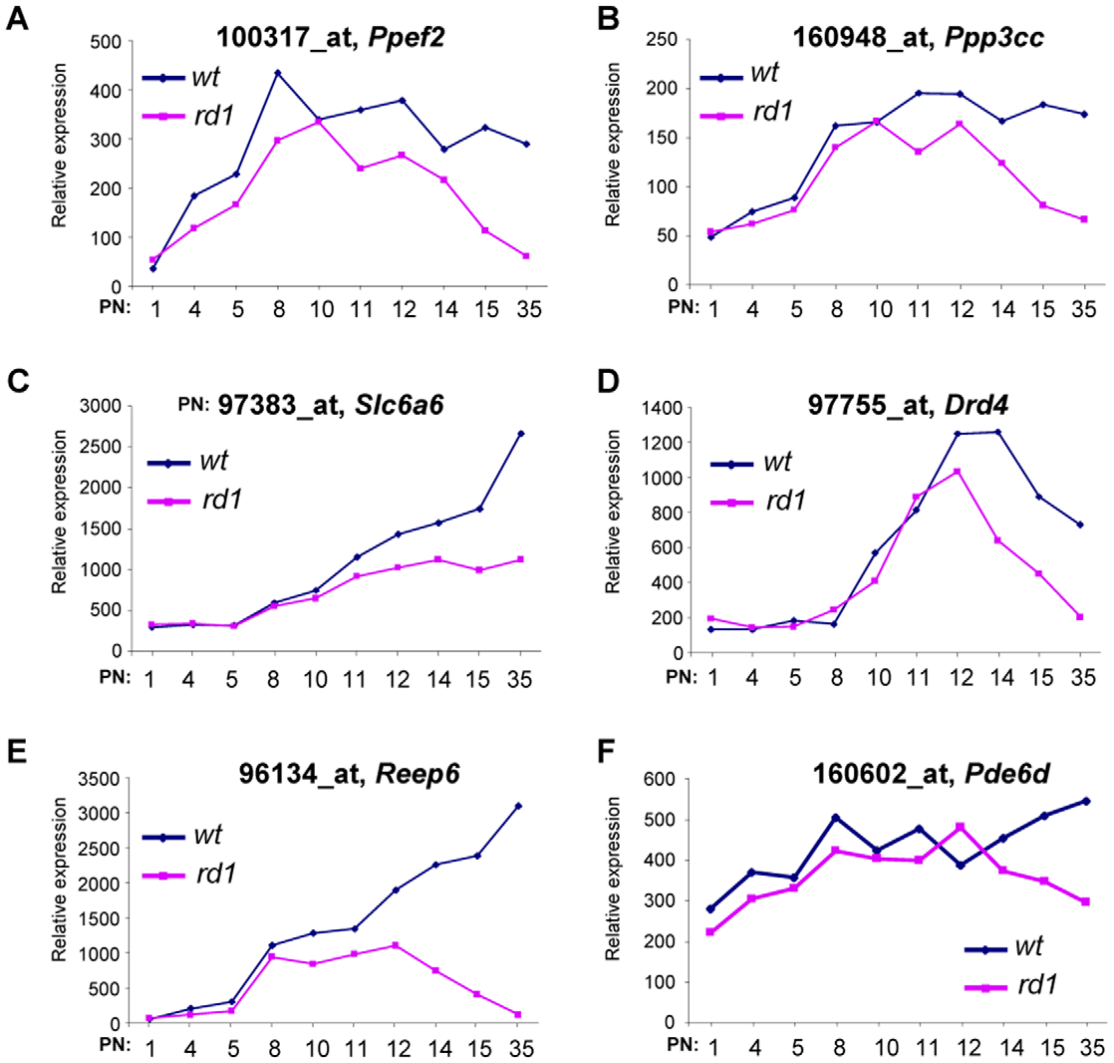
Kinetics of expression of the orthologous of a subset of the target genes in the *wild-type* and *rd1* retina between PN1 and PN35. (A) *Pepf2*, (B) *Ppp3cc*, (C) *Slc6a6*, (D) *Drd4*, (E) *Reep6* and (F) *Pde6d*. PN, post-natal day.

The presence of the *NXNL1* and five photoreceptor specific genes (*ABCA4*, *RDH12*, *CNGB1*, *SAG* and *CUCA1C*), whose mutations cause inherited retinal degenerations, in the list of 156 down-regulated probesets with high MI suggests that MI identifies events linked to photoreceptor dysfunction or degeneration. These 156 probesets were examined for their expression in a genetic model of rod photoreceptor degeneration, the *rd1* mouse. In this model the recessive mutation of *Pde6b* leads to a rapid degeneration of rods [41]. By 35 post-natal days (PN), all the rods (half of the neurons of the retina) are lost [42]. We performed a global analysis of the *rd1* neural retina using Affymetrix technology (see methods). We examined the expression of 44 mouse orthologues of genes down-regulated in RD indentified by MI analysis and for which the expression value was >20 (Table 4). It should be noticed that their ratios outer retina/neural retina has an average of 1.19. Since the outer retina is the portion of the neural retina containing the photoreceptors, it indicates that this list is enriched for genes rather specifically expressed by photoreceptors in the retina [43]. Within this dataset, the expression in the *wild-type* (*wt*) and *rd1* retina at PN35 of a subset of 25 orthologous genes down-regulated in RD and having the highest MI score was compared to 25 up-regulated genes with the highest FDR value (Figure S2). We found that the average ratio *wt*/*rd1* is 2.89 for genes down-regulated in RD and 0.85 for those that are up-regulated, suggesting a certain degree of conservation in the pathways involved in RD and photoreceptor degeneration in the *rd1* model. The kinetic of expression of the protein phosphatase *Ppef2* increases from PN1 to PN8 for both *wt* and *rd1* during the process of normal post natal maturation of the retina. From PN11 to PN35 the difference of expression between the *rd1* versus the *wt* mouse is correlated with rod degeneration (Figure 6A), as for the protein phosphatase *Ppp3cc* (Figure 6B). This is even observed earlier, from PN10 for the receptor accessory gene *Reep6* (Figure 6C), and from PN12 for the dopamine receptor *Drd4* (Figure 6D). The expression of the Taurine transporter *Slc6a6* in the mutant retina is lagging behind *wt* from PN11 to PN35 (Figure 6E) and from PN14 for the phosphodiesterase *Pde6d* (Figure 6F). The expression of these target genes, down-regulated in RD, correlated with the degeneration of rod photoreceptors in the *rd1* mouse. The comparison with the *rd1* transcriptome supports the fact that the mutual information identifies target genes related to the process of photoreceptor degeneration or dysfunction in RD.

**Table 4:** A Subset of down-regulated genes identified by mutual information analysis.

Gene Name	Gene Symbol	Affymetrix	OR/NR
ATP-binding cassette, sub-family A, member 4	*ABCA4*	97730_at	1.57
Adducin 1 (alpha)	*ADD1*	94535_at	1.01
A kinase (PRKA) anchor protein 1	*AKAP1*	97368_at	1.15
Adaptor-related protein complex 1, gamma 1 subunit	*AP1G1*	103242_at	1.20
Rho GTPase activating protein 5	*ARHGAP5*	92247_at	1.22
ATPase family, AAA domain containing 2B	*ATAD2B*	93426_at	1.43
Carbonic anhydrase XIV	*CA14*	98079_at	0.63
Calcium channel, voltage-dependent, beta 2 subunit	*CACNB2*	100757_at	1.58
Calsequestrin 1	*CASQ1*	102426_at	0.99
Dopamine receptor D4	*DRD4*	97755_at	1.07
Estrogen-related receptor beta	*ESRRB*	100301_at	1.27
Enhancer of zeste homolog 1 (Drosophila)	*EZH1*	100486_at	1.71
UDP-N-acetyl-alpha-D-galactosamine	*GALNT2*	97553_at	0.95
Heat shock protein 90kDa alpha (cytosolic), class A member 1	*HSP90AA1*	95282_at	1.24
Kinesin family member 1B	*KIF1B*	160871_at	1.25
Leucine-rich repeats and calponin homology (CH) domain containing 4	*LRCH4*	104151_at	1.18
Membrane-bound transcription factor peptidase	*MBTPS1*	95754_at	0.88
Myosin, light chain 6	*MYL6*	97542_at	1.04
NADH dehydrogenase (ubiquinone) 1 alpha subcomplex	*NDUFA4*	160477_at	0.95
Poly(A) binding protein interacting protein 2B	*PAIP2B*	96158_at	1.06
Phosphodiesterase 6D	*PDE6D*	160602_at	1.32
6-phosphofructo-2-kinase/fructose-2,6-biphosphatase 2	*PFKFB2*	98329_at	1.44
Phospholipase A2, group V	*PLA2G5*	101328_at	1.17
Polymerase (DNA-directed), epsilon 4	*POLE4*	95121_at	0.90
Protein phosphatase, EF-hand calcium binding domain 2	*PPEF2*	100317_at	1.77
Protein phosphatase 1, catalytic subunit, gamma isoform	*PPP1CC*	101482_at	1.21
Protein phosphatase 3 (formerly 2B), catalytic subunit, gamma isoform	*PPP3CC*	160948_at	1.25
PTC7 protein phosphatase homolog (S. cerevisiae)	*PPTC7*	99503_at	0.81
PRP4 pre-mRNA processing factor 4 homolog B (yeast)	*PRPF4B*	102017_at	1.29
Parvalbumin	*PVALB*	96719_i_at	0.86
Renal tumor antigen MOK	*RAGE*	104166_at	1.14
Receptor accessory protein 6	*REEP6*	96134_at	1.65
Retinal G protein coupled receptor	*RGR*	95331_at	0.94
Ribosomal RNA processing 1 homolog B (S. cerevisiae)	*RRP1B*	93130_at	1.20
S-antigen; retina and pineal gland (arrestin)	*SAG*	94150_at	1.16
Splicing factor, arginine/serine-rich 3	*SFRS3*	101004_f_at	1.58
Solute carrier family 25	*SLC25A4*	93084_at	0.84
Solute carrier family 4, sodium bicarbonate cotransporter, member 7	*SLC4A7*	93471_at	1.82
Solute carrier family 6	*SLC6A6*	97383_at	1.09
Serine palmitoyltransferase, long chain base subunit 2	*SPTLC2*	100893_at	1.09
Synovial sarcoma, X breakpoint 2 interacting protein	*SSX2IP*	96723_f_at	1.02
Syntaxin binding protein 1	*STXBP1*	97983_s_at	1.36
T-complex-associated-testis-expressed 3	*TCTE3*	99134_at	1.16
Ubiquinol-cytochrome c reductase binding protein	*UQCRB*	95472_f_at	0.90

OR, outer retina. NR, neural retina.

## Discussion

**Figure 7:**
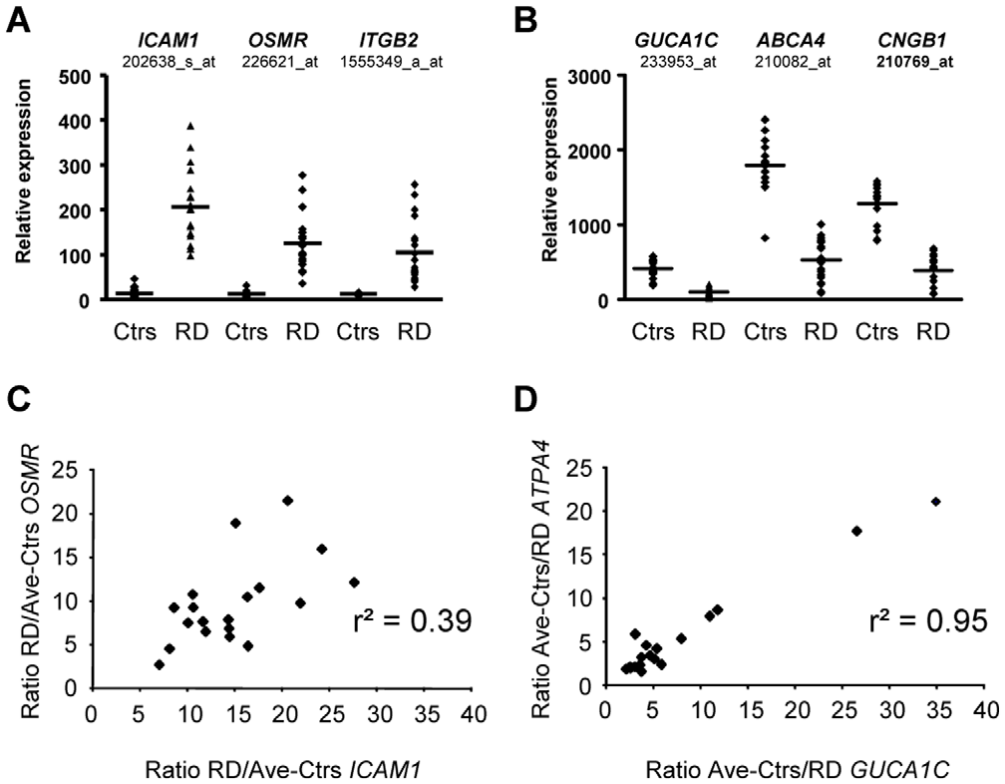
Dynamic of the change in expression. (A) Relative expression of three upregulated probesets selected by FDR but ignored by MI. *ICAM1*: intercellular adhesion molecule 1, *OSMR*: oncostatin M receptor, *ITGB2*, integrin, beta 2. The histograms display controls (Ctrs) and retinal detachment (RD) for each specimen. The horizontal line is the average. (B) Relative expression of thee downregulated probesets selected by MI but ignored by FDR. *GUCA1C*: guanylate cyclase activator 1C, *ABCA4*: ATP-binding cassette, *CNGB1*: cyclic nucleotide gated channel beta 1. (C) Graph displaying the ratios retinal detachments versus the average value of controls (RD/Ctrs) for *ICAM1* and *OSMR*. **D.** Graph displaying the ratios of the average value of controls versus retinal detachments versus (Ctrs/RD) for *GUCA1C* and *ATPA4*.

The mechanism involved in retinal detachment at the genome level has only been addressed to date on animal models of the pathology [44], [45], [46]. These models involve the artificial detachment of the retina using a surgical procedure and do not address the human situation, with an accidentally detached retina. This artificial setting most likely explains why the IL6 and the Aryl hydrocarbon receptor oxidative stress pathways highlighted in a rat study [44] are not reproduced the clinical specimens studied here (Table S3). This difference seriously questions the physiopathological relevance of such animal models. The human tissues have only been studied *in vitro* [47]. To our knowledge, it is the first time that the transcriptome of human clinical specimens have been analyzed providing results that can be directly interpreted in medical terms. Transcriptomics analysis of human retinal detachment reveals the existence of two pathophysiological processes: 1) an inflammatory response that is most likely implicated in 2) the damage to the photoreceptor cells. In the retina, inflammatory mediators might also be acting in a paracrine manner to affect neighboring cells and triggering the PVR process [48]. These two aspects were captured by two successive but finally complementary analyses of the microarray data. The classic FDR method identified genes that globally participate in the inflammatory response. This method is particularly suited for analyzing the transcriptome of animal models of disease. One reason is that these animals are often inbred strains in which the variability within an experimental group is considered as non-informative and consequently averaged. In the case of surgical specimens, each individual displays a retinal transcriptome that not only reflects the disease but is also modulated by the activity of modifier genes in the genome and by differences in environmental exposure, or treatment. We have used this interindividual variability as a guide to identifying co-regulated genes using MI, a method developed to detect dependencies between variables [14]. MI highlights the existence of a coordinated program linked to the degeneration of photoreceptor cells. The two processes are correlated as seen by the fact that *HLA-C*, an up-regulated gene involved in the inflammatory response, is anti-correlated to down-regulated genes *PKD2L1* and *SLCO4A1*, markers of photoreceptor death. What could explain why the analysis of the same set of expression data by these two methods should reveal two different but complementary aspects of the pathophysiological process? A critical difference in the nature of these two events is likely translated into a alternative dynamics of the gene expression changes. The inflammatory response is massive and results from the induction of genes from an unstimulated state. This in turn explains the high ratio (Fold change) observed when comparing RD versus controls and favours the statistical significance and the identification using FDR, even when the three stages of the disease were considered. For example *ICAM1*, *OSMR* and *ITGB2*, all involved in inflammation are identified by the FDR, but not the MI method (Figure 7A). On the opposite side, photoreceptor degeneration results in the loss of expression (called here down-regulation) of photoreceptor-enriched mRNAs, an on/off situation with an amplitude limited by the expression level at start, i.e. in normal condition (controls). This limitation of the amplitude is likely to impair the statistical significance and results in the low number of down-regulated genes of at least two-fold as identified by the FDR method. For example, *GUCA1C*, *ABCA4* and *CNGB1* were all detected by the MI, but not the FDR method (Figure 7B). This ordered process is most likely variable from one individual to the other in relation to the stage of the disease, genetic susceptibility and environmental conditions (e.g. medication). This explains why some of the photoreceptor-specific genes do not achieve statistical significance, but also implies that their expression is coordinated within each individual specimen. Here the ratio (Average Ctrs)/RD of *GUCA1C* and *ATPA4* for all RD patients are correlated (r^2^ = 0.95, Figure 7D). Conversely, the immune response involves the activation of a cascade of genes expressed by cells invading the retina and emitting signals that result in non linear variations, translated into less coordination and into induction of the various members of this signalling pathway within each individual. This finally results in a loose co-regulation that is detrimental to the mutual information analysis. Here the ratios RD/(Average Ctrs) for *ICAM1* and *OSMR* are not correlated (r^2^ = 0.39, Figure 7C).

From a mechanistic point of view, one of the biomarkers *HLA-C* measures the state of inflammatory response, and another, *SLCO4A1*, scores photoreceptor degeneration in all three subgroups of RD. The anion transporter *SLCO4A1* is largely distributed in the body but is expressed in a rod-dependent manner in the mouse retina (result not shown), so it is likely that its down-regulation is related to a disturbance of efflux of ions into the detached retina. The TRP channel family member *PKD2L1* is involved in sensing acidic taste [49]. Its down-regulation in the late class of RD may be related to an extended damage to the retina (Fig 4B). Its role in the retina is unknown but its expression is rather eye-specific (UniGene Hs.159241) and may represent a candidate gene for inherited retinal dystrophies, as TRPM1 was found to be involved in congenital stationary night blindness [50] (Figure 5).

From a therapeutic point of view, identifying these new targets implicated either in photoreceptor cell degeneration and/or in inflammation response is a key step towards achieving a current challenge in RD management, the development of adjuvant therapies. We show here that photoreceptor degeneration during RD proceeds through mechanisms shared with a model of inherited photoreceptor degeneration, the *rd1* mouse. This suggests that neuroprotective strategies developed for treating retinitis pigmentosa to prevent photoreceptor cell death could be used as adjuvant therapy for RD, this includes for example, the use of the RdCVF protein [40]. As shown in table 4 and Fig. 6, the loss of expression of the following genes was observed similarly in both conditions: dopamine receptor D4 (*Drd4*), receptor expression enhancing protein 6 (*Reep6*), taurine transporter *Slc6a6*, protein phosphatase EF-hand calcium binding domain 2 (*Ppef2*) and protein phosphatase 3 (*Ppp3cc*). For target genes expressed by the photoreceptor themselves, such as *DRD4* and *PPEF2* [51], [52], [53], it is difficult to conclude using the data reported here whether the loss of their expression is correlated to directly to the loss of photoreceptors or to the mechanism of cell death. Down-regulation of *PPEF2* gene could both reflect and/or accelerate the degenerative process, and the use of DRD4 agonists may be beneficial.

*PPP3CC* encodes for the gamma subunit of the protein phosphatases 3 (PPP3), one of the major serine/threonine phosphatases in the brain. PPP3 dephosphorylates the TAU protein that is involved in forming neurotoxic deposits in the brain of patients suffering from Alzheimer's disease [54], [55]. The phosphorylation of TAU is a hallmark of the process leading to the formation of neurofibrillary tangle made of hyperphosphorylated TAU [56]. Interestingly, we reported recently that TAU is hyperphosphorylated in the retinal degeneration mouse model carrying an inactivation of the *Nxnl1* gene encoding the cone viability factor RdCVF [57]. Here, we show that *Ppp3cc* expression is down-regulated in the *rd1* retina (Fig 7B). As PPP3CC participates in TAU homeostasis, its decrease during retinal degeneration, either due to inherited condition (*rd1*) or to retinal detachment (RD), may induce TAU hyperphosphorylation and further accelerate neuronal death through TAU fibrillization. Inhibition of TAU phosphorylation could henceforth represent means for slowing down photoreceptor degeneration during RD using either molecules that stimulate PPP3 phosphatase activity or other inhibitors of TAU phosphorylation such as RdCVFL [57].

Another lead is provided by *SLC6A6* expression. Taurine is an amino acid that plays essential roles during the development of the central nervous system, and in the maintenance of mature neural tissues. Taurine inhibits light-induced lipid peroxidation and protects rod photoreceptors [58]. Taurine transporter knockout mice (*slc6a6*-/- or Taut-/- mice) exhibit decreased taurine levels in many tissues and an age-dependent degeneration of photoreceptor cells by apoptosis leading to blindness at an early age. [59]. During RD, the long-lasting separation of the photoreceptors from the underlying RPE is associated with a decrease of *Slc6a6* expression that could participate in their loss through apoptosis. The over-expression of *Slc6a6* could represent a way of slowing down photoreceptor degeneration during RD.

In summary, our work sheds new light on the pathophysiological events that occur following human retinal detachment and raises new targets for a pharmacologic adjuvant therapy to be combined with the current surgical management of RD. Further studies on animal models of RD will be needed before strategies using the target genes identified here can be applied to RD therapeutic management in clinical trials.

## Materials and Methods

### Patients and control specimens

A total of 56 consecutive patients with rhegmatogenous retinal detachment (RD), and for which pars plana vitrectomy associated with large peripheral retinectomy was considered necessary, were investigated in five departments of ophthalmology. Indications for retinectomy in eyes with RD were failure of retinal reattachment by conventional methods and/or severe proliferative vitreoretinopathy (PVR). Patients with a history of diabetes, trauma or any associated concurrent eye condition such as vitreous haemorrhage, glaucoma, infection or uveitis were excluded. The severity of PVR was graded according to the updated classification of the Retina Society Terminology Committee [22]. Data concerning prior surgeries, duration of retinal detachment and severity of PVR were documented. Patients included in the study had given their informed consent and the study complied with the Declaration of Helsinki. The patients from UK have given their written consent. For the French patients, the Comité consultative pour la protection des personnes dans la recherche biomédicale advised that since the specimens are res nullius (Code civil art. 539), a written consent is not required. The ethic committees (Comité consultative pour la protection des personnes dans la recherché biomédicale) and the British committee (Moorsfields Eye Hospital) Moorsfields Eye Hospital) approved the study. Retinal samples were obtained through standard three-port 20-Gauge pars plana vitrectomy: a conventional surgical procedure aimed at removing the vitreous to allow retinal surgery. Vitreous was first entirely removed and an intra-ocular diathermy was performed in the area of retinectomy to prevent hemorrhages. Retinectomy was performed after complete posterior hyaloid dissection and membrane peeling of PVR. The vitreotome was then rinsed by aspiration of 10 ml of BSS® (Balanced Salt Solution isotonic to the tissues of the eye, Alcon Laboratories, Inc., Fort Worth, TX). The aspiration line of the vitreotome was then connected to a 10 ml syringe and the retinectomy performed using the manual suction. Retinal fragments of detached retina were collected into the syringe filled with BSS®. The fragments were rapidly pelleted at the bottom of the syringe, the buffer discarded and retinal samples were immersed in Guanidium chloride solution (see below), homogenized and sent to the laboratory for RNA purification. Among the 56 samples, only 19 were retained for further analyses either due to insufficient quantities of RNA (less than 5 µg for 35 samples) or to partial RNA degradation (2 samples). Out of 19 residual patients, 14 were men and 5 were women (Table 1) with a mean age of 52 years (range 23–74 years). Human retinal specimens used as controls were post-mortem specimens collected within 12 hours following death of patients with no past medical history of eye disease or diabetes and obtained at the Cornea Bank of Amsterdam (The Netherlands). After corneal removal for transplantation purposes, the remaining eye tissue was immediately dissected in BSS®, and the retina cautiously separated from both vitreous body and retinal pigment epithelium (RPE). Only peripheral neural retina was considered for further analyses in order to match the surgical specimens. Nineteen samples were collected from 19 eyes representing 17 patients. Sex ratio was 12 men/7 women with a mean age of 61 years (range 25–78 years) (Table 1).

### RNA purification and quantitative RT-PCR analysis

Total RNAs were purified using the cesium chloride centrifugation [60]. Absence of RNA degradation was assessed by RNA denaturing agarose gel electrophoresis and the quantity of RNA extracted was determined by optical density measurement. Samples with RNA degradations or yielding less than 5 µg were excluded.

cDNAs were synthesized by reverse transcription using random hexamers (pdN6) according to standard protocols. All primer pairs were validated by sequencing the PCR products (Table S4). Quantitative RT-PCR was performed on a LightCycler instrument (Roche-Diagnostics, Indianapolis, IN) with SYBR Green I, according to the manufacturer's instructions. Quantitative RT-PCR analyses were performed in duplicate on 8 samples of each group. Cycling conditions were as follows: initial denaturation at 95°C for 2 min, followed by 40 cycles of denaturation at 95°C for 0 sec, annealing for 5 sec (see Table S4 for annealing temperatures) and elongation at 72°C for 15 sec. Melting curve analysis was performed as follows: denaturation at 95°C for 2 min, annealing at 65°C for 2 min, followed by a gradual increase (0.1°C/sec) in temperature to 95°C. RT-PCR efficiency for each pair of primers was measured by calculating the slope of a linear regression graph using manufacturer's instructions. For each experiment, crossing points were calculated by the LightCycler Data Analysis Program (LightCycler-3.5 Software). Target gene expressions were normalized with respect to β-actin mRNA. Amplification products (10 µl) were validated by agarose gel electrophoresis (data not shown). For statistical analyses, unpaired Student's t-test was used to compare mRNAs expression in RD samples and controls. Values of p<0.05 were considered to be significant.

### Microarray procedures

For human specimen 2 µg of purified total RNA were used. For mouse tissues, 5 µg from pools of five neural retinas (5 individuals) from *wild-type* (C57BL6@N) or *rd1* (C3H/He@N) mice were dissected at 11 post natal (PN) days 1, 4, 5, 8, 10, 11, 12 14, 15 and 35. The RNA were used to generate biotin-labeled cRNA probes hybridized to the Affymetrix Human genome U133 plus 2.0 array or Mouse genome U74abc. Quality Control (QC) performed using RReportGenerator (Data S1).

### Bioinformatics analysis

Differential gene expression in the transcriptome of RD was determined on GCRMA normalized data [61] using moderated t-test [62] and local false discovery rate (FDR) [36] or mutual information (MI). MI is considered here as a statistical generalization of the correlation For Mutual information analysis, we consider the relevance of a variable subset 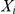 to predict a target 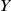 be revealed by the mutual information 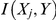 [63], [64]. The mutual information is a symmetric and non-negative measurement and is null if and only if the two subsets are statistically independent. As in [65], the mutual information estimation is based on the method of Kraskov et al. [66] that was improved here through bootstrap to reduce the variance of the mutual information estimator. Indeed, the final estimation is the mean of the mutual information computed over 50 sampling collections of the dataset (random sampling with replacement, equal size). Nevertheless, considering the number of samples, we are far from the asymptotic conditions of the mutual information estimator and furthermore the mutual information is homogeneous with the entropy, which means it is not normalized. This can be a problem to define relevant thresholds. So, as the correlation is more useful than the covariance, we defined a normalized mutual information 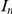 for each target subset 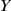 so that:

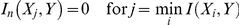
: according to the number of variables we explore, we can consider that the variable with the lowest mutual information with 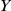 and 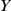 are independent, in other words the value is the bias of the estimator.

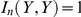
: the target is fully informative about itself.

Finally, 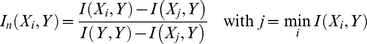


Protein-protein interactions were analyzed using the STRING database containing known and predicted, physical and functional protein-protein interactions as described previously [57]. STRING in protein mode was used and only interactions with high confidence levels (>0.7) were kept. We used the DAVID functional annotation tool to identify the enriched gene ontology (GO) terms within the gene lists [38]. In particular, we used the functional annotation clustering procedure at “high” classification stringency using all 3 categories of GO simultaneously as input. Resultant clusters represent groups of similar GO ontologies that were selected based on a cluster-enrichment score [38] higher than 1.0 and a minimum FDR value for the highest enriched GO term per group of <50%.

The normalized mutual information was used in three different ways. First, the normalized mutual information between every variable (expression pattern of a gene) and the class was computed to make first a selection of relevant genes. Second, among these relevant genes, an iterative procedure to build a group of complementary variables was implemented by selecting each time the variable that yields a group with the best normalized mutual information for the class [65], [67]. At last, for every variable in the final group, we evaluated the variables that have the highest normalized mutual information value and are likely to be implicated in the same pathway. All data is MIAME compliant and that the raw data has been deposited in a MIAME compliant database at Gene Expression Omnibus with the accession numbers GSE28133. Protein-protein interactions were analyzed as described in Fridlich et al. [57].

## Supporting Information

Figure S1 **Selection of the mutual information values.** The graph plotted the mutual information versus the absolute value of the correlation coefficient. The green dots correspond to the 266 probesets selected based on their mutual information.
(PPT)

Figure S2 **Expression of the orthologous of a subset of the target genes in the** ***wild-type*** **and** ***rd1*** **retina at PN35**. (A) The expression of the down-regulated genes with the highest MI. (B) The expression of the up-regulated genes with the highest FDR.
(PPT)

Table S1 L**ist of probesets selected with false discovery rate and mutual information.** (http://lbgi.igbmc.fr/RetinalDetachment/).
(DOC)

Table S2 **Enrichment in gene ontology terms for probesets selected with false discovery rate and mutual information**. (http://lbgi.igbmc.fr/RetinalDetachment/).
(DOC)

Table S3 **Modified expression of Stress-response genes in rat versus human retinal detachment.**
(DOC)

Table S4 **Sequences and annotations of the primers used for quantitative RT-PCR.**
(DOC)

Data S1 **Affymetrix Batch Quality Control using RReportGenerator and R.**
(PDF)
