# Supplementary material for: Transcriptomic Analysis of Human Retinal Detachment Reveals Both Inflammatory Response and Photoreceptor Death
Source: PLoS One. 2011 Dec 9;6(12):e28791. doi: 10.1371/journal.pone.0028791 (PMC3235162; doi:10.1371/journal.pone.0028791)
Supplement: Figure S1 — Selection of the mutual information values. The graph plotted the mutual information versus the absolute value of the correlation coefficient. The green dots correspond to the 266 probesets selected based on their mutual information. (PPT) [file pone.0028791.s001.ppt]

## Slide 1
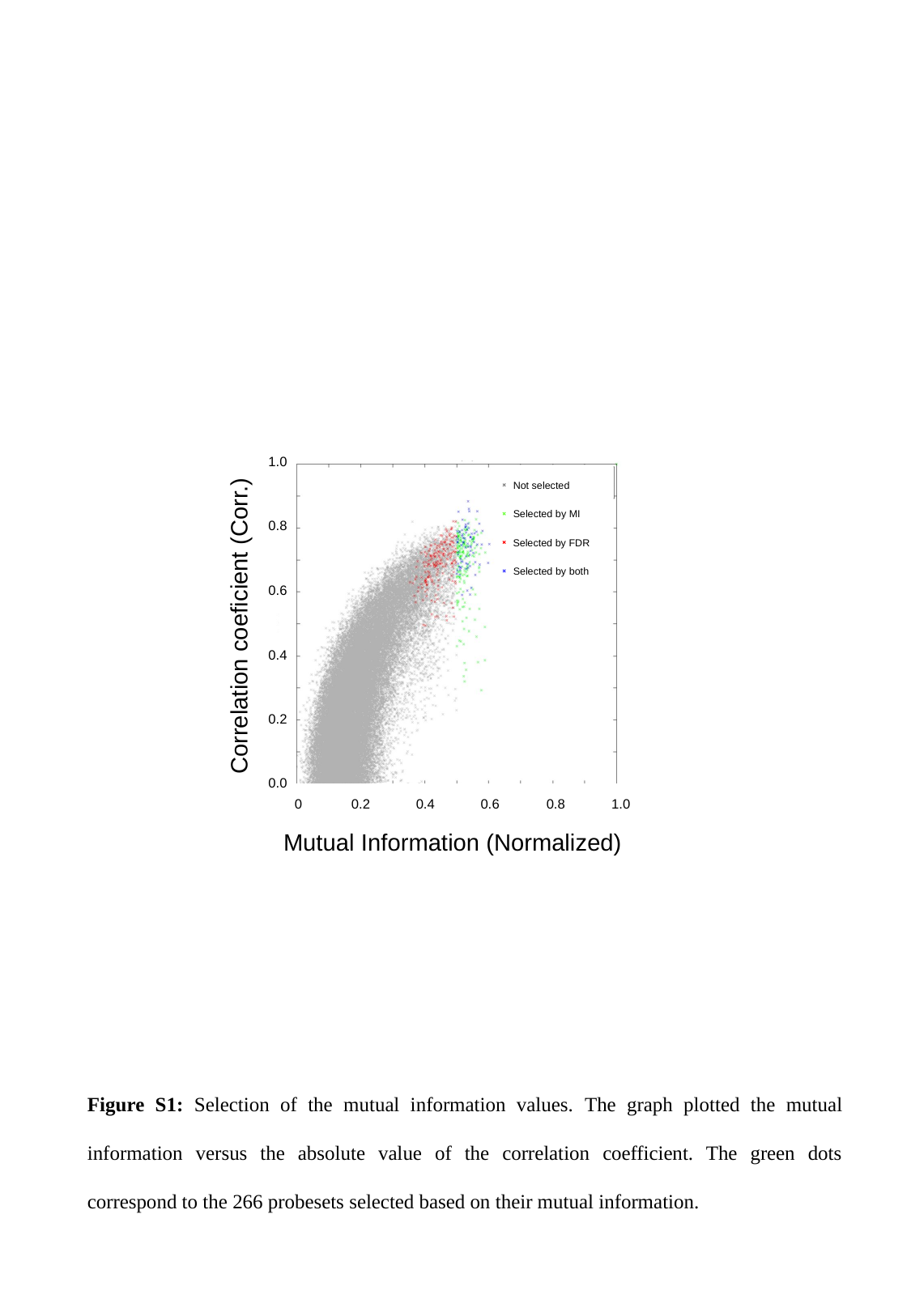

1.0
Not selected
Selected by MI
0.8
Selected by FDR
Selected by both
0.6
Correlation coeficient (Corr.)
0.4
0.2
0.0
0
0.2
0.4
0.6
0.8
1.0
Mutual Information (Normalized)
Figure S1: Selection of the mutual information values. The graph plotted the mutual information versus the absolute value of the correlation coefficient. The green dots correspond to the 266 probesets selected based on their mutual information.
