# Supplementary material for: Transcriptomic Analysis of Human Retinal Detachment Reveals Both Inflammatory Response and Photoreceptor Death
Source: PLoS One. 2011 Dec 9;6(12):e28791. doi: 10.1371/journal.pone.0028791 (PMC3235162; doi:10.1371/journal.pone.0028791)
Supplement: Figure S2 — Expression of the orthologous of a subset of the target genes in the wild-type and rd1 retina at PN35. (A) The expression of the down-regulated genes with the highest MI. (B) The expression of the up-regulated genes with the highest FDR. (PPT) [file pone.0028791.s002.ppt]

## Slide 1
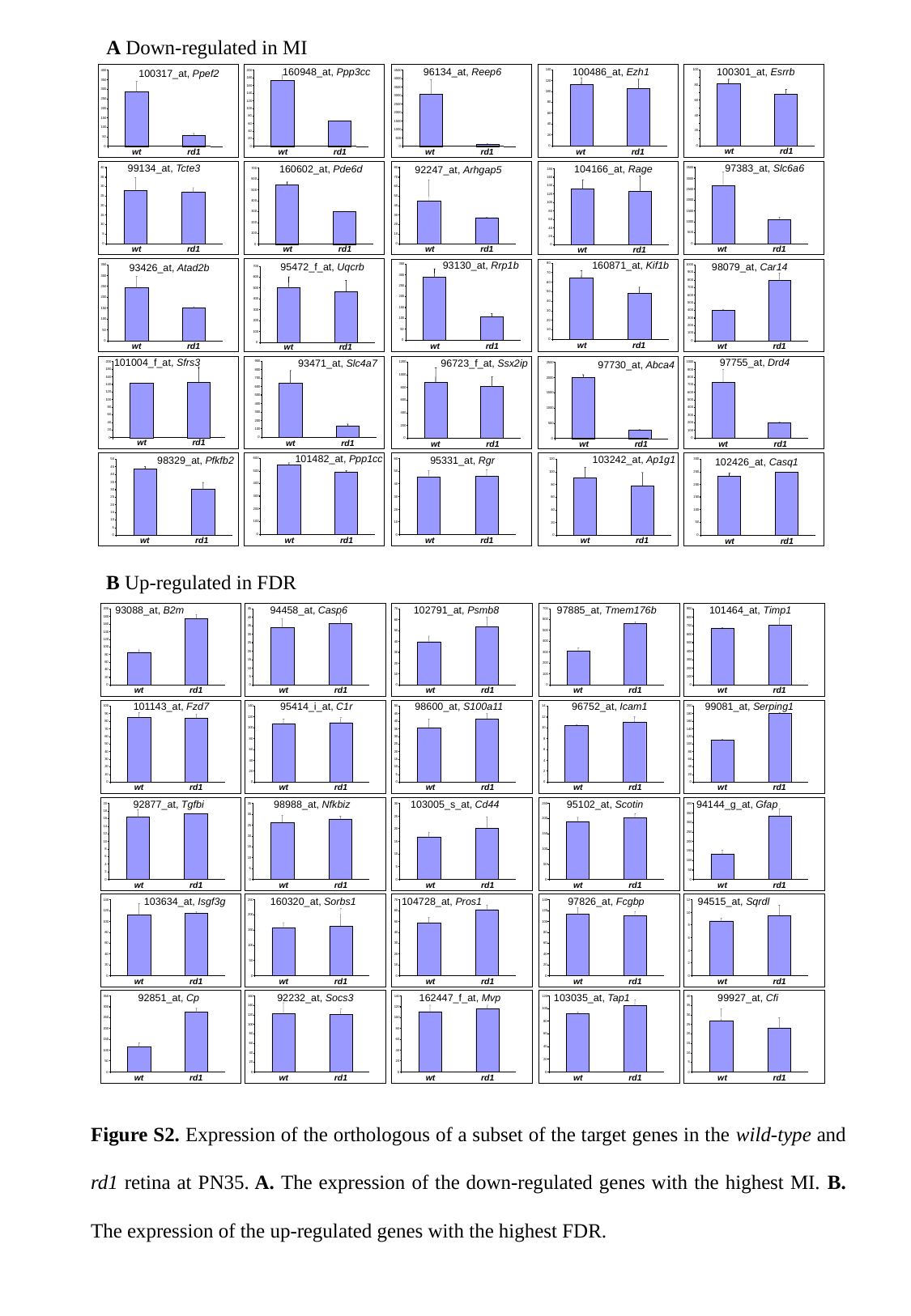

A Down-regulated in MI
96134_at, Reep6
4500
4000
3500
3000
2500
2000
1500
1000
500
0
wt
rd1
100301_at, Esrrb
100
80
60
40
20
0
wt
rd1
160948_at, Ppp3cc
200
180
160
140
120
100
80
60
40
20
0
wt
rd1
100486_at, Ezh1
140
120
100
80
60
40
20
0
wt
rd1
100317_at, Ppef2
400
350
300
250
200
150
100
50
0
wt
rd1
99134_at, Tcte3
40
35
30
25
20
15
10
5
0
wt
rd1
97383_at, Slc6a6
3500
3000
2500
2000
1500
1000
500
0
wt
rd1
104166_at, Rage
180
160
140
120
100
80
60
40
20
0
wt
rd1
160602_at, Pde6d
700
600
500
400
300
200
100
0
wt
rd1
92247_at, Arhgap5
80
70
60
50
40
30
20
10
0
wt
rd1
93130_at, Rrp1b
350
300
250
200
150
100
50
0
wt
rd1
160871_at, Kif1b
80
70
60
50
40
30
20
10
0
wt
rd1
95472_f_at, Uqcrb
700
600
500
400
300
200
100
0
wt
rd1
98079_at, Car14
1000
900
800
700
600
500
400
300
200
100
0
wt
rd1
93426_at, Atad2b
350
300
250
200
150
100
50
0
wt
rd1
101004_f_at, Sfrs3
200
180
160
140
120
100
80
60
40
20
0
wt
rd1
97755_at, Drd4
1000
900
800
700
600
500
400
300
200
100
0
wt
rd1
96723_f_at, Ssx2ip
1200
1000
800
600
400
200
0
wt
rd1
93471_at, Slc4a7
900
800
700
600
500
400
300
200
100
0
wt
rd1
97730_at, Abca4
2500
2000
1500
1000
500
0
wt
rd1
101482_at, Ppp1cc
600
500
400
300
200
100
0
wt
rd1
103242_at, Ap1g1
120
100
80
60
40
20
0
wt
rd1
98329_at, Pfkfb2
50
45
40
35
30
25
20
15
10
5
0
wt
rd1
95331_at, Rgr
60
50
40
30
20
10
0
wt
rd1
102426_at, Casq1
300
250
200
150
100
50
0
wt
rd1
B Up-regulated in FDR
93088_at, B2m
200
180
160
140
120
100
80
60
40
20
0
wt
rd1
94458_at, Casp6
45
40
35
30
25
20
15
10
5
0
wt
rd1
102791_at, Psmb8
70
60
50
40
30
20
10
0
wt
rd1
97885_at, Tmem176b
700
600
500
400
300
200
100
0
wt
rd1
101464_at, Timp1
900
800
700
600
500
400
300
200
100
0
wt
rd1
101143_at, Fzd7
100
90
80
70
60
50
40
30
20
10
0
wt
rd1
95414_i_at, C1r
140
120
100
80
60
40
20
0
wt
rd1
98600_at, S100a11
50
45
40
35
30
25
20
15
10
5
0
wt
rd1
96752_at, Icam1
14
12
10
8
6
4
2
0
wt
rd1
99081_at, Serping1
200
180
160
140
120
100
80
60
40
20
0
wt
rd1
92877_at, Tgfbi
20
18
16
14
12
10
8
6
4
2
0
wt
rd1
98988_at, Nfkbiz
35
30
25
20
15
10
5
0
wt
rd1
103005_s_at, Cd44
30
25
20
15
10
5
0
wt
rd1
95102_at, Scotin
250
200
150
100
50
0
wt
rd1
94144_g_at, Gfap
400
350
300
250
200
150
100
50
0
wt
rd1
103634_at, Isgf3g
140
120
100
80
60
40
20
0
wt
rd1
160320_at, Sorbs1
250
200
150
100
50
0
wt
rd1
104728_at, Pros1
70
60
50
40
30
20
10
0
wt
rd1
97826_at, Fcgbp
140
120
100
80
60
40
20
0
wt
rd1
94515_at, Sqrdl
12
10
8
6
4
2
0
wt
rd1
92851_at, Cp
350
300
250
200
150
100
50
0
wt
rd1
92232_at, Socs3
160
140
120
100
80
60
40
20
0
wt
rd1
162447_f_at, Mvp
140
120
100
80
60
40
20
0
wt
rd1
103035_at, Tap1
120
100
80
60
40
20
0
wt
rd1
99927_at, Cfi
40
35
30
25
20
15
10
5
0
wt
rd1
Figure S2. Expression of the orthologous of a subset of the target genes in the wild-type and rd1 retina at PN35. A. The expression of the down-regulated genes with the highest MI. B. The expression of the up-regulated genes with the highest FDR.
