# Supplementary material for: Transcriptomic Analysis of Human Retinal Detachment Reveals Both Inflammatory Response and Photoreceptor Death
Source: PLoS One. 2011 Dec 9;6(12):e28791. doi: 10.1371/journal.pone.0028791 (PMC3235162; doi:10.1371/journal.pone.0028791)
Supplement: Table S2 — Enrichment in gene ontology terms for probesets selected with false discovery rate and mutual information. (http://lbgi.igbmc.fr/RetinalDetachment/). (DOC) [file pone.0028791.s004.doc]

**Table S2: Enrichment in gene ontology terms for probesets selected with false discovery rate and mutual information** (http://lbgi.igbmc.fr/RetinalDetachment/).
