# Supplementary material for: Transcriptomic Analysis of Human Retinal Detachment Reveals Both Inflammatory Response and Photoreceptor Death
Source: PLoS One. 2011 Dec 9;6(12):e28791. doi: 10.1371/journal.pone.0028791 (PMC3235162; doi:10.1371/journal.pone.0028791)
Supplement: Table S3 — Modified expression of Stress-response genes in rat versus human retinal detachment. (DOC) [file pone.0028791.s005.doc]

| **Gene Name (rat)** | **Fold Change (FC)** | | **Human**  **Gene Symbol** | **Human Probeset#** | **FC** |
| --- | --- | --- | --- | --- | --- |
|  | **rat_RD 3d*** | **rat_RD 7d*** |  |  | **Human_RD** |
| *Interleukin-6/STAT pathway* | | | | | |
| Interleukin-6 | 22.6 | 35.9 | IL6 | 205207_at | 1.67 |
| Interleukin-6 alpha receptor | 8.2 | 6.5 | IL6R | 205945_at | 1.44 |
| Interleukin-6 beta receptor (gp130) | 4.1 | 7.6 | IL6ST | 212195_at | 1.33 |
| CNTF | 7.8 | 9.0 | CNTF | 208597_at | 1.01 |
| STAT1 | 14.6 | 87.1 | STAT1 | 200887_s_at | 4.03 |
| STAT2 | 1.6 | 13.9 | STAT2 | 225636_ats | 1.28 |
| STAT3 | 2.5 | 5.3 | STAT3 | 208991_at | 2.72 |
| STAT4 | 14.7 | 6.9 | STAT4 | 206118_at | 1.07 |
| STAT5 | 1.0 | 1.5 | STAT5A | 203010_at | 1.24 |
| *Transforming growth factor-beta pathway* | | | | | |
| Transforming growth factor-beta1 | 4.0 | 38.7 | TGFB1 | 203085_s_at | 5.49 |
| Transforming growth factor-beta2 | 4.9 | 1.0 | TGFB2 | 228121_at | 1.26 |
| Transforming growth factor-beta3 | 6.7 | 53.2 | TGFB3 | 209747_at | 1.30 |
| Smad1 | 1.8 | 1.3 | SMAD1 | 210993_s_at | 4.28 |
| Smad2 | 1.5 | 1.3 | SMAD2 | 203075_at | 0.98 |
| Smad3 | 2.3 | 2.6 | SMAD3 | 218284_at | 1.81 |
| Smad4 | 1.3 | 1.4 | SMAD4 | 202527_s_at | 0.98 |
| Smad5 | 1.4 | 2.3 | SMAD5 | 225219_at | 1.04 |
| Smad7 | 1.3 | 1.4 | SMAD7 | 204790_at | 1.63 |
| Smad8 | 1.8 | 2.3 | SMAD9 | 206320_s_at | 1.05 |
| *Aryl hydrocarbon receptor-oxidative stress response genes* | | | | | |
| Cytochrome P450 | 6.3 | 17.1 | CYP1A1 | 205749_at | 0.99 |
| NAD(P)H:quinone oxidoreductase 1 | 14.6 | 9.1 | NQO1 | 201468_s_at | 2.10 |
| Aldehyde dehydrogenase 3 | 18.6 | 6.2 | ALDH3A1 | 205623_at | 1.12 |
| UDP-glucuronosyltransferase | 11.4 | 3.4 | UGT1A6 | 206094_x_at | 1.00 |
| Glutathione transferase | 5.5 | 6.2 | GSTA1 | 215766_at | 0.92 |

**Table S3: Modified expression of Stress-response genes in rat versus human retinal detachment.** Fold change of detached retina versus controls.* Data according to Zacks et al. [1]. 3d and 7d, three and seven days after surgical detachment.

1. Zacks DN, Han Y, Zeng Y, Swaroop A (2006) Activation of signaling pathways and stress-response genes in an experimental model of retinal detachment. Invest Ophthalmol Vis Sci 47: 1691-1695.
