# Supplementary material for: Transcriptomic Analysis of Human Retinal Detachment Reveals Both Inflammatory Response and Photoreceptor Death
Source: PLoS One. 2011 Dec 9;6(12):e28791. doi: 10.1371/journal.pone.0028791 (PMC3235162; doi:10.1371/journal.pone.0028791)
Supplement: Table S4 — Sequences and annotations of the primers used for quantitative RT-PCR. (DOC) [file pone.0028791.s006.doc]

| **Gene Name** | **Gene Symbol** | **Forward (1) and**  **reverse (2)**  **Primer sequence** | **Annealing**  **Temperature** | **Product**  **Length (bp)** |
| --- | --- | --- | --- | --- |
| AlphaB crystallin | *CRYAB* | 1:CACCTGTTGGAGTCTGATCTTTTCCC  2:AGACGCCAATACCAATGGTCCAG | 58 | 231 |
| Alpha-smooth muscle actin | *ACTA2* | 1:tgacarcaggaaggacctctatgc  2:aaggcccggcttcatcgtattc | 58 | 242 |
| ASK1 | *MAP3K5* | 1:cagggggvtgaagaaccgaaactaaaatgg  2:ggcaaacatccagtgcggcttgatgttatg | 57 | 240 |
| Beta actin | *ACTB* | 1:agtcctgtggcatccacgaaac  2:tacttgcgctcaggaggagcaatg | 58 | 202 |
| CD4 | *CD4* | 1: taaccgaggtcccttgtcccaagttc  2:tcctctggtaacagattctgccacc | 58 | 234 |
| CD68 | *CD68* | 1:tcgagtcatgtacacaacccagg  2:aaggacacattgtactccaccgc | 58 | 213 |
| c-fos | *FOS* | 1:cgttgtgaagaccatgacaggagg  2:ctccgcttggagtgtatcagtcag | 58 | 175 |
| Cyclin A1 | *CCNA1* | 1:tagggctgctaactgcaaatgg  2:ccggtgtctacttcatacacatcc | 57 | 250 |
| Cyclin D1 | *CCND1* | 1:agaagctgtgcatctacaccgac  2:catggagggcggattggaaatg | 58 | 242 |
| Cytochrome oxidase | *COX4I1* | 1:tagttcccctaataatcggtgccccc  2:acctgctaggtgtaaggagaagatgg | 58 | 218 |
| GFAP | *GFAP* | 1 : ATCACCATTCCCGTGCAGACCTTCTC  2 : TGCCTCACATCACATCCTTGTGCTC | 58 | 175 |
| GLAST | *SLC1A3* | 1:ttcacgcagtcatcgtcttgccac  2:aaagcctcatagagggcagtcccatc | 58 | 244 |
| Glutamine synthetase | *GLUL* | 1:ggaggccattgagaaactaagcaagc  2:agaggggcgacgatcttcaaag | 58 | 217 |
| Intercellular adhesion molecule 1 | *ICAM1* | 1:ccccagaaggagtgatttttctatcggcac  2:tgcaaacaggacaagaggacaaggc | 58 | 246 |
| L-cone Opsin | *OPN1LW* | 1:tgcatcatcccactcgct  2:gacgcagtacgcaaagat | 62 | 155 |
| Matrix MetalloProteinase 2 | *MMP2* | 1:aggatgacatcaagggcattcaggag  2:cgtcacagtccgccaaatgaac | 58 | 191 |
| M-cone opsin | *OPN1MW* | 1:tgcatcaccccactcagc  2:gaagcagaatgccaggac | 62 | 155 |
| Ornithine decarboxylase | *ODC1* | 1:ggaacgggcgaaagagctaaatatcg  2:gtccaacgctgggttgattacg | 58 | 238 |
| PCNA | *PCNA* | 1:aggcactcaaggacctcatcaac  2:tatccgcgttatcttcggcccttag | 58 | 243 |
| Phosducin | *PDC* | 1:agcaagaaggagattctcaggc  2:ggtgcatatcctgcatac | 54 | 175 |
| RdCVF | *NXNL1* | 1:tgtaatcccagcacttggggag  2:acaccgggctaacttttaattttcgtagag | 56 | 120 |
| Rhodopsin | *RHO* | 1:cggaggtcaacaacgagtcttttgtc  2:gtgaagatgtagaatgccacgctg | 58 | 244 |
| S-cone Opsin | *OPN1SW* | 1:agctgtaacggataacttcgtcttcgg  2:agacgccaataccaatggtccag | 58 | 211 |
| Synaptophysin | *SYP* | 1:tgtactttgatgcacccacctgc  2:atgccgatgagctaactagccac | 58 | 240 |
| Tissue Inhibitor Metalloproteinase 1 | *TIMP1* | 1:ggatacttccacaggtcccacaac  2:gcagtttgcaggggatggataaacag | 58 | 208 |

**Table S4: Sequences and annotations of the primers used for quantitative RT-PCR**.
