# Supplementary material for: Transcriptomic Analysis of Human Retinal Detachment Reveals Both Inflammatory Response and Photoreceptor Death
Source: PLoS One. 2011 Dec 9;6(12):e28791. doi: 10.1371/journal.pone.0028791 (PMC3235162; doi:10.1371/journal.pone.0028791)
Supplement: Data S1 — Affymetrix Batch Quality Control using RReportGenerator and R. (PDF) [file pone.0028791.s007.pdf]

Data S1:

Affymetrix Batch Quality Control using RReportGenerator and R

March 22, 2011

This document was generated by Analysis Type File '*automAffyQC2.Rnw*', Version: 1.2.9, a protocol for automated QC analysis for Affymetrix expression array data.

The aim of this report is to provide information on multiple QC aspects for a set of mircoarrays. The interpretation of QC-plots should be done with care since this may lead to delicate decisions. For further information about the various plots in this report please look at the references section. Analysis protocol written by wolfgang.raffelsberger (at) igbmc.fr, LGBI, IGBMC (Strasbourg, France).

QC analysis of 38 cel-files of type 'HG-U133.Plus.2' with 54675 probesets each, found in path :

`/data2/g3/wrTest/RNG209/`

page 3
Both plots give a resume of the original PM (perfect match) values, either as boxplot or as (kernel) density estimate for the signal distribution (similar to Bioconductor-packages simpleaffy and affyQCReport).

page 4
This figure shows the QC plot from the simpleaffy package. For this purpose the 3' to 5' ratio for spiked-in and control genes (triangles for b-Actin and squares for GAPDH) are considered. The filled (blue) dot with the vertical line (heading to 0) represents the scaling factor. Finally, the percentage of 'present calls' and value of average background are shown in red on the left side. Note that the order of arrays is inversed (compared to other plots in this report).

page 5
This pair of graphs shows the RLE (top) and NUSE (bottom) plots from the affyPLM package. The RLE compares for each probeset (across all arrays) its expression value against its median across all arrays. The NUSE plot shows the standard errors for each probe-set (variability intra), standardized across all arrays.

page 6
This page shows false color images for the RLE residuals of all probes (from the affyPLM package, with red intensities corresponding to positive residuals and blue to negative residuals). Note, that smaller deviating zones have little impact on the overal results. For further information, examples and details see e.g. chapter 3 by Bolstad in book by Gentleman *et al.*

page 7
MA plots (affyPLM package) of each array against a synthetic median array (probe-wise medians of all arrays in project). The red line represents a lowess fit to the scatter plot and may help indicating non-linear relationships.

page 8
Top: RNA degradation plot shows the average intensity with respect to the sorted 5' to 3' position of the probes in the target-sequence. Depending on the type of microarray specific patterns can be observed (see also references Bolstad, Gentleman et al). Boxplots (center) and distribution estimates (bottom) for data resulting from GCRMA normalization.

page 9
Top: Similarity between GCRMA summarized samples measured as Euclidean distance : Heatmap of distance values.
Bottom: Dendrogram from hierarchical clustering (with p-values estimated by boostraping (reference Suzuki 2004)

page 10
Principal components of GCRMA summarized samples. The main graphic shows the values of the first and second principal component for each of the samples. The smaller plots show the 2nd vs. 3rd as well as the 1st vs. 3rd component, the bargraph at the bottom indicates the amount of total variance captured by the individual principal components.

page 11 : References

Overview of Arrays Used

array index	(short) sample names	/ initial file−name
1 ...	CTR10	.. CTR10_U133_2.CEL.gz
2 ...	CTR11	.. CTR11_U133_2.CEL.gz
3 ...	CTR12	.. CTR12_U133_2.CEL.gz
4 ...	CTR13	.. CTR13_U133_2.CEL.gz
5 ...	CTR14	.. CTR14_U133_2.CEL.gz
6 ...	CTR15	.. CTR15_U133_2.CEL.gz
7 ...	CTR16	.. CTR16_U133_2.CEL.gz
8 ...	CTR17	.. CTR17_U133_2.CEL.gz
9 ...	CTR18	.. CTR18_U133_2.CEL.gz
10 ...	CTR19	.. CTR19_U133_2.CEL.gz
11 ...	CTR1	.. CTR1_U133_2.CEL.gz
12 ...	CTR2	.. CTR2_U133_2.CEL.gz
13 ...	CTR3	.. CTR3_U133_2.CEL.gz
14 ...	CTR4	.. CTR4_U133_2.CEL.gz
15 ...	CTR5	.. CTR5_U133_2.CEL.gz
16 ...	CTR6	.. CTR6_U133_2.CEL.gz
17 ...	CTR7	.. CTR7_U133_2.CEL.gz
18 ...	CTR8	.. CTR8_U133_2.CEL.gz
19 ...	CTR9	.. CTR9_U133_2.CEL.gz
20 ...	RD10	.. RD10_U133_2.CEL.gz
21 ...	RD11	.. RD11_U133_2.CEL.gz
22 ...	RD12	.. RD12_U133_2.CEL.gz
23 ...	RD13	.. RD13_U133_2.CEL.gz
24 ...	RD14	.. RD14_U133_2.CEL.gz
25 ...	RD15	.. RD15_U133_2.CEL.gz
26 ...	RD16	.. RD16_U133_2.CEL.gz
27 ...	RD17	.. RD17_U133_2.CEL.gz
28 ...	RD18	.. RD18_U133_2.CEL.gz
29 ...	RD19	.. RD19_U133_2.CEL.gz
30 ...	RD1	.. RD1_U133_2.CEL.gz
31 ...	RD2	.. RD2_U133_2.CEL.gz
32 ...	RD3	.. RD3_U133_2.CEL.gz
33 ...	RD4	.. RD4_U133_2.CEL.gz
34 ...	RD5	.. RD5_U133_2.CEL.gz
35 ...	RD6	.. RD6_U133_2.CEL.gz
36 ...	RD7	.. RD7_U133_2.CEL.gz
37 ...	RD8	.. RD8_U133_2.CEL.gz
38 ...	RD9	.. RD9_U133_2.CEL.gz

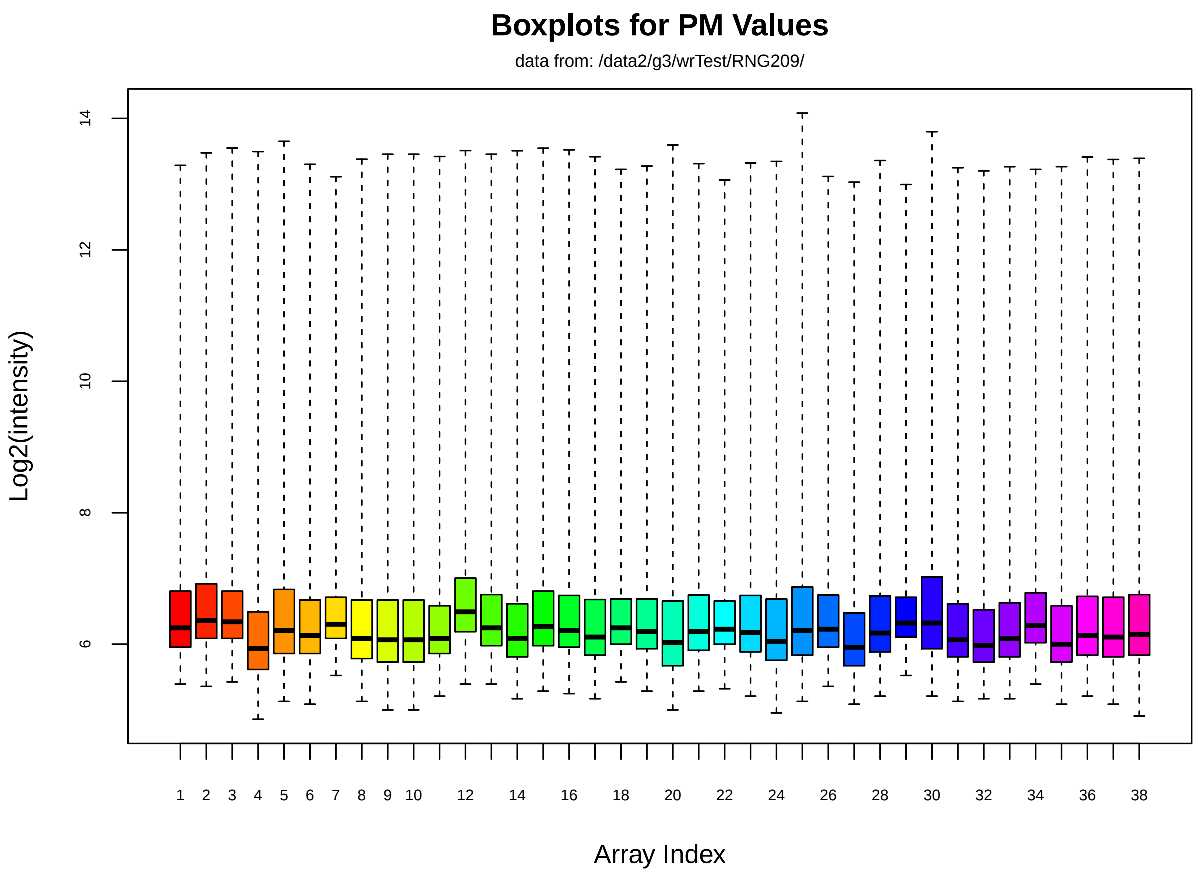
Boxplots for PM Values
data from: /data2/g3/wrTest/RNG209/
Log2(intensity)
Array Index

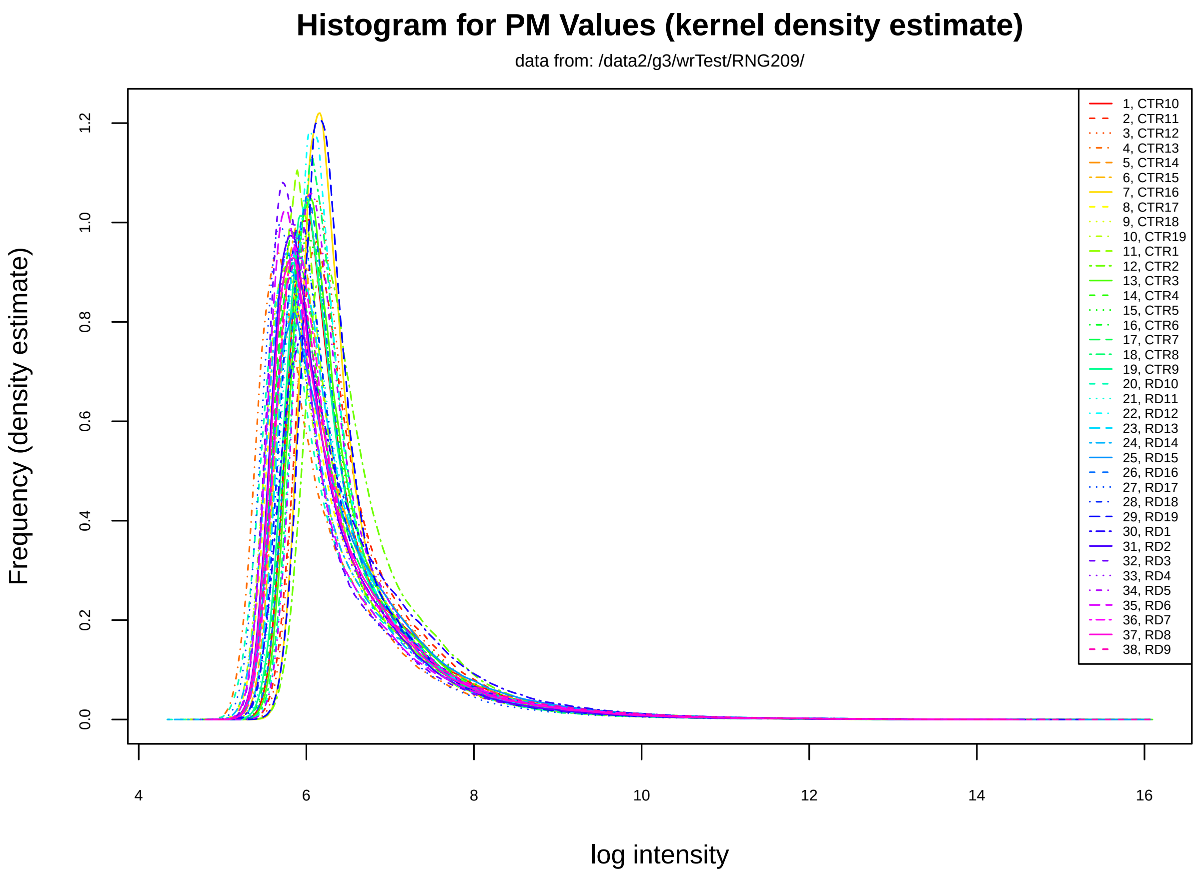
Histogram for PM Values (kernel density estimate)
data from: /data2/g3/wrTest/RNG209/
Frequency (density estimate)
log intensity
1, CTR10
2, CTR11
3, CTR12
4, CTR13
5, CTR14
6, CTR15
7, CTR16
8, CTR17
9, CTR18
10, CTR19
11, CTR1
12, CTR2
13, CTR3
14, CTR4
15, CTR5
16, CTR6
17, CTR7
18, CTR8
19, CTR9
20, RD10
21, RD11
22, RD12
23, RD13
24, RD14
25, RD15
26, RD16
27, RD17
28, RD18
29, RD19
30, RD1
31, RD2
32, RD3
33, RD4
34, RD5
35, RD6
36, RD7
37, RD8
38, RD9

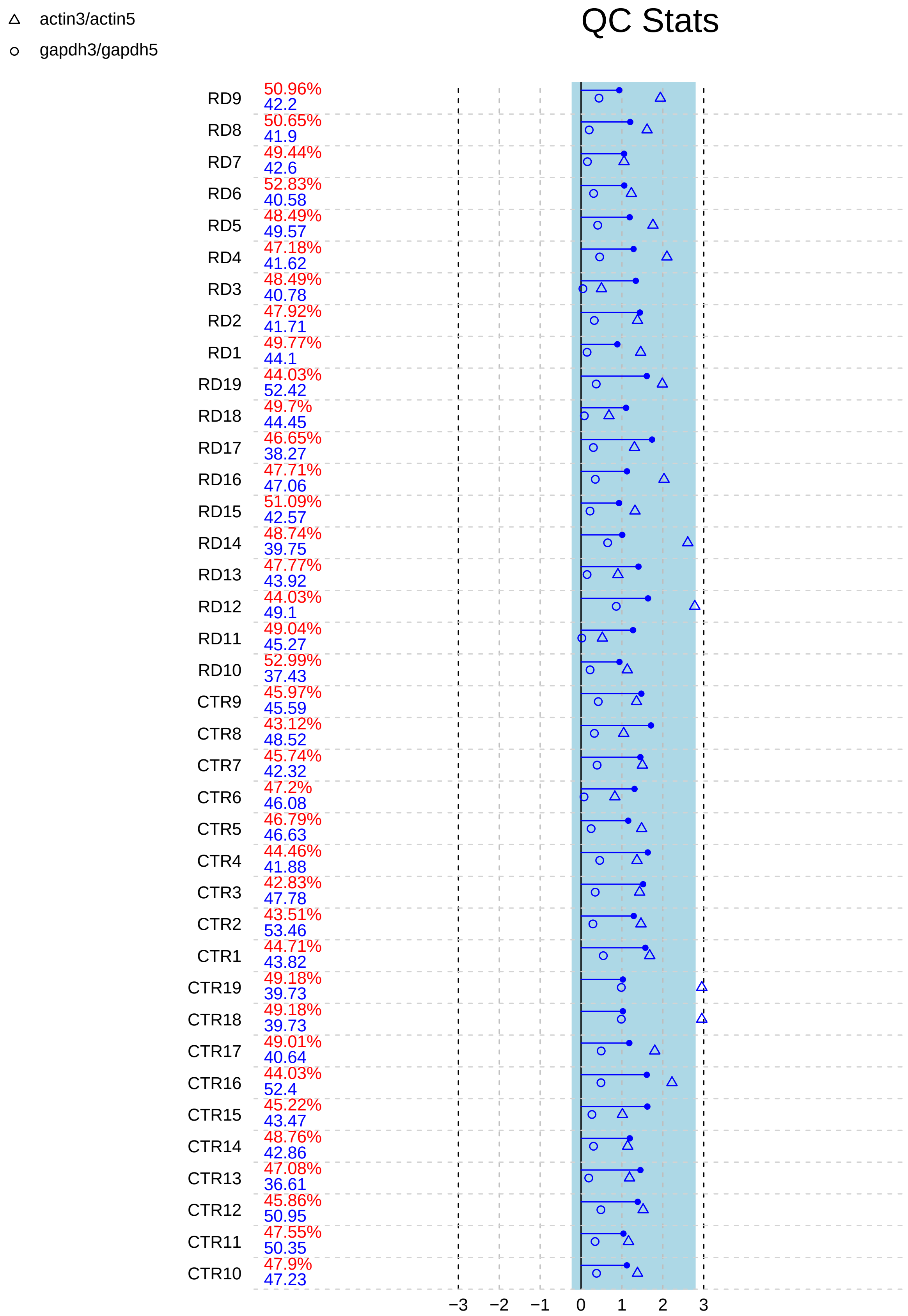
actin3/actin5
gapdh3/gapdh5
QC Stats
RD9 50.96% 42.2
RD8 50.65% 41.9
RD7 49.44% 42.6
RD6 52.83% 40.58
RD5 48.49% 49.57
RD4 47.18% 41.62
RD3 48.49% 40.78
RD2 47.92% 41.71
RD1 49.77% 44.1
RD19 44.03% 52.42
RD18 49.7% 44.45
RD17 46.65% 38.27
RD16 47.71% 47.06
RD15 51.09% 42.57
RD14 48.74% 39.75
RD13 47.77% 43.92
RD12 44.03% 49.1
RD11 49.04% 45.27
RD10 52.99% 37.43
CTR9 45.97% 45.59
CTR8 43.12% 48.52
CTR7 45.74% 42.32
CTR6 47.2% 46.08
CTR5 46.79% 46.63
CTR4 44.46% 41.88
CTR3 42.83% 47.78
CTR2 43.51% 53.46
CTR1 44.71% 43.82
CTR19 49.18% 39.73
CTR18 49.18% 39.73
CTR17 49.01% 40.64
CTR16 44.03% 52.4
CTR15 45.22% 43.47
CTR14 48.76% 42.86
CTR13 47.08% 36.61
CTR12 45.86% 50.95
CTR11 47.55% 50.35
CTR10 47.9% 47.23
−3 −2 −1 0 1 2 3

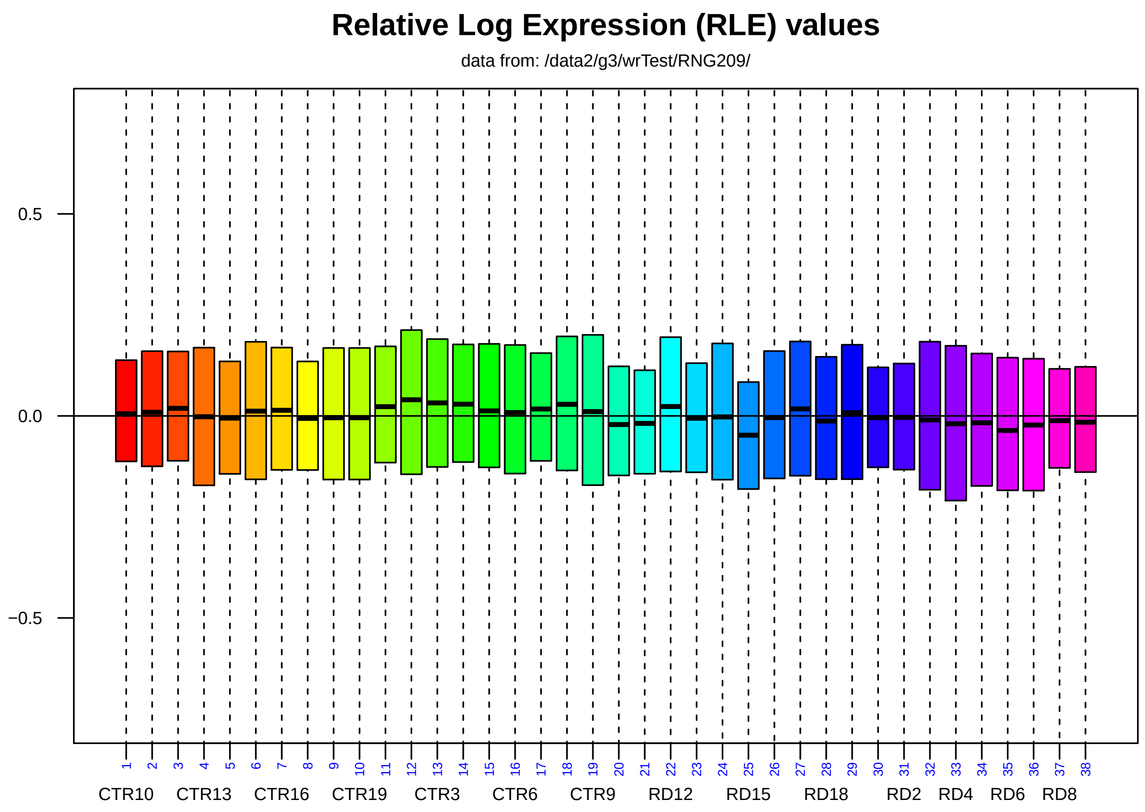
Relative Log Expression (RLE) values
data from: /data2/g3/wrTest/RNG209/
0.5
0.0
−0.5
1 2 3 4 5 6 7 8 9 10 11 12 13 14 15 16 17 18 19 20 21 22 23 24 25 26 27 28 29 30 31 32 33 34 35 36 37 38
CTR10 CTR13 CTR16 CTR19 CTR3 CTR6 CTR9 RD12 RD15 RD18 RD2 RD4 RD6 RD8

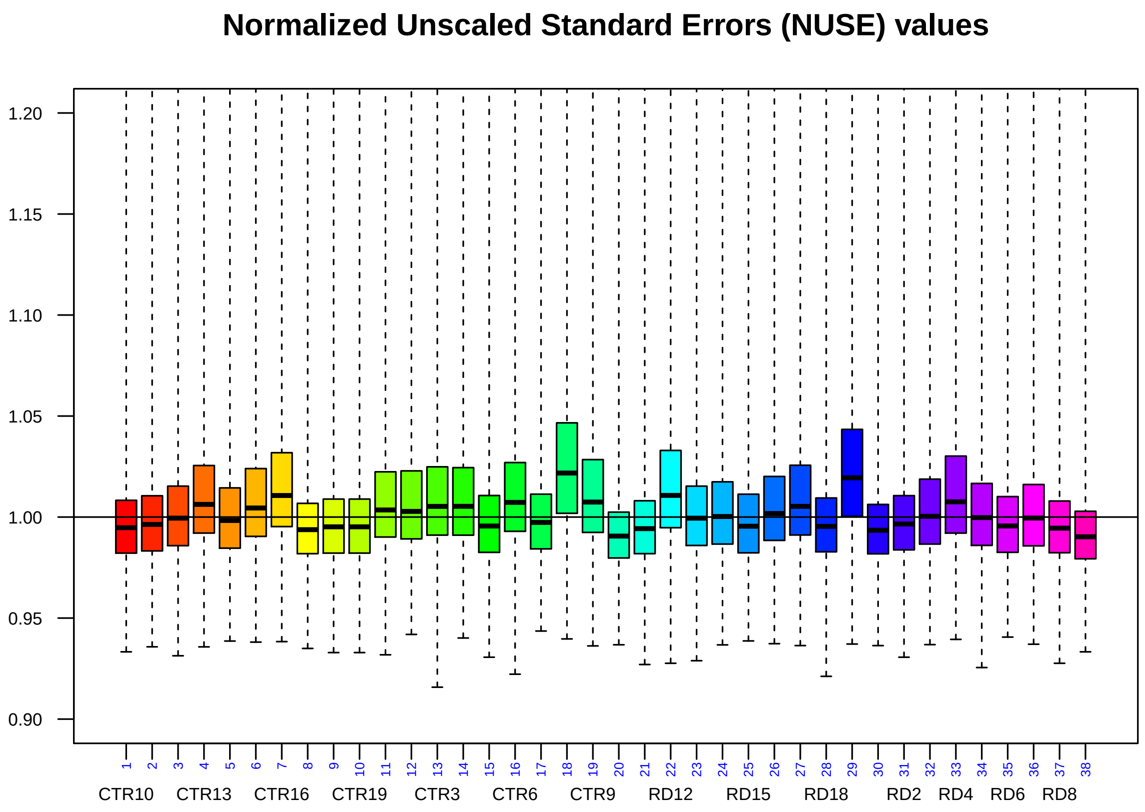
Normalized Unscaled Standard Errors (NUSE) values
1.20
1.15
1.10
1.05
1.00
0.95
0.90
1 2 3 4 5 6 7 8 9 10 11 12 13 14 15 16 17 18 19 20 21 22 23 24 25 26 27 28 29 30 31 32 33 34 35 36 37 38
CTR10 CTR13 CTR16 CTR19 CTR3 CTR6 CTR9 RD12 RD15 RD18 RD2 RD4 RD6 RD8

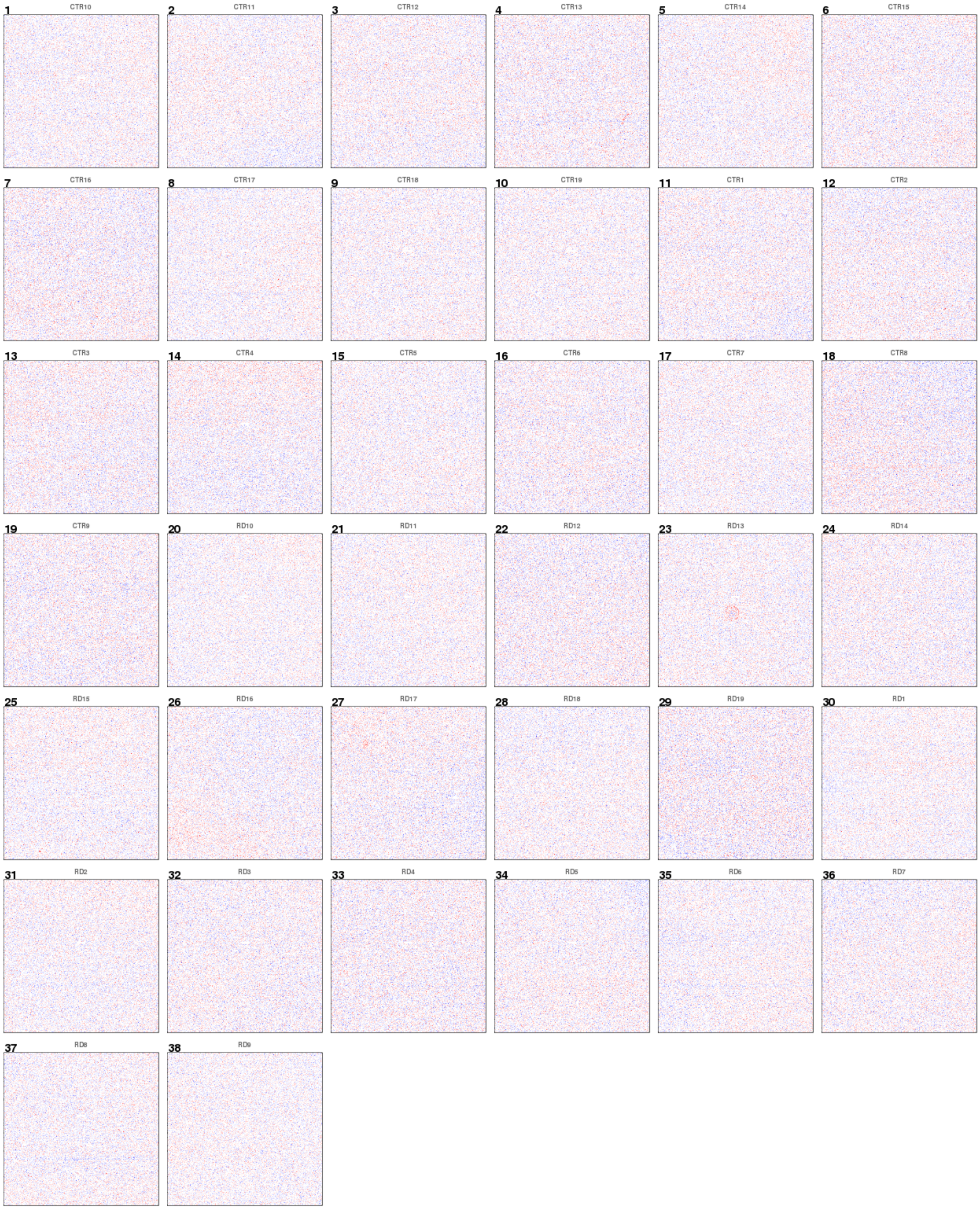
1 CTR10
2 CTR11
3 CTR12
4 CTR13
5 CTR14
6 CTR15
7 CTR16
8 CTR17
9 CTR18
10 CTR19
11 CTR1
12 CTR2
13 CTR3
14 CTR4
15 CTR5
16 CTR6
17 CTR7
18 CTR8
19 CTR9
20 RD10
21 RD11
22 RD12
23 RD13
24 RD14
25 RD15
26 RD16
27 RD17
28 RD18
29 RD19
30 RD1
31 RD2
32 RD3
33 RD4
34 RD5
35 RD6
36 RD7
37 RD8
38 RD9

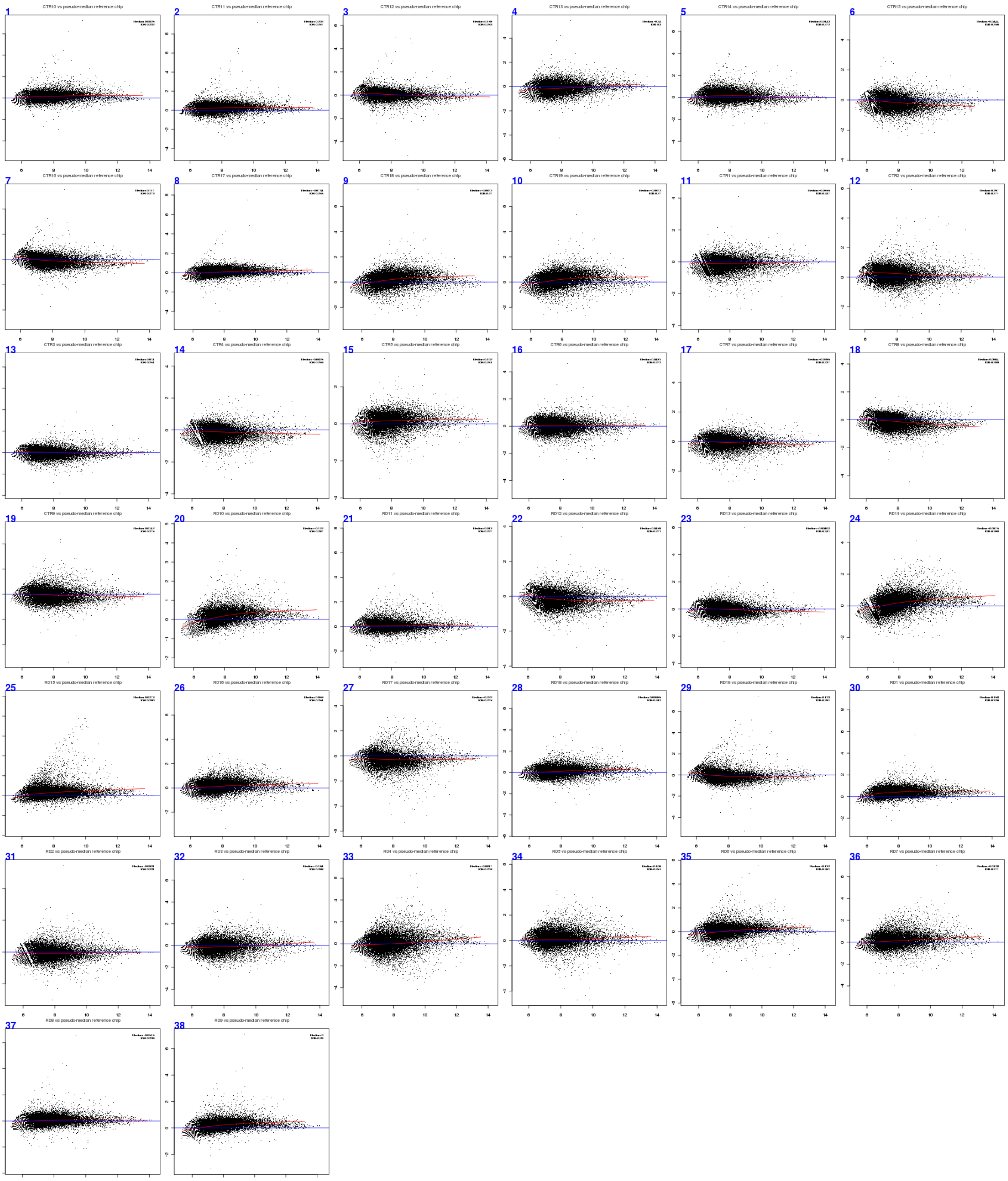
1 CTR10 vs pseudo-median reference chip
2 CTR11 vs pseudo-median reference chip
3 CTR12 vs pseudo-median reference chip
4 CTR13 vs pseudo-median reference chip
5 CTR14 vs pseudo-median reference chip
6 CTR15 vs pseudo-median reference chip
7 CTR16 vs pseudo-median reference chip
8 CTR17 vs pseudo-median reference chip
9 CTR18 vs pseudo-median reference chip
10 CTR19 vs pseudo-median reference chip
11 CTR1 vs pseudo-median reference chip
12 CTR2 vs pseudo-median reference chip
13 CTR3 vs pseudo-median reference chip
14 CTR4 vs pseudo-median reference chip
15 CTR5 vs pseudo-median reference chip
16 CTR6 vs pseudo-median reference chip
17 CTR7 vs pseudo-median reference chip
18 CTR8 vs pseudo-median reference chip
19 CTR9 vs pseudo-median reference chip
20 RD10 vs pseudo-median reference chip
21 RD11 vs pseudo-median reference chip
22 RD12 vs pseudo-median reference chip
23 RD13 vs pseudo-median reference chip
24 RD14 vs pseudo-median reference chip
25 RD15 vs pseudo-median reference chip
26 RD16 vs pseudo-median reference chip
27 RD17 vs pseudo-median reference chip
28 RD18 vs pseudo-median reference chip
29 RD19 vs pseudo-median reference chip
30 RD1 vs pseudo-median reference chip
31 RD2 vs pseudo-median reference chip
32 RD3 vs pseudo-median reference chip
33 RD4 vs pseudo-median reference chip
34 RD5 vs pseudo-median reference chip
35 RD6 vs pseudo-median reference chip
36 RD7 vs pseudo-median reference chip
37 RD8 vs pseudo-median reference chip
38 RD9 vs pseudo-median reference chip

RNA degradation plot

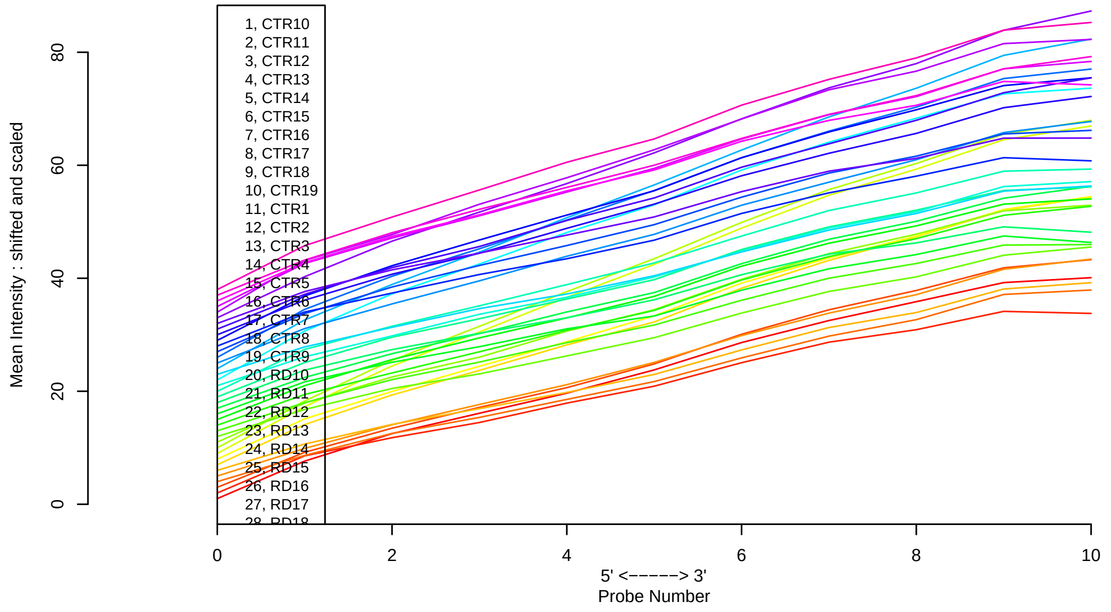

Boxplots for GCRMA Processed Signal Intensity

data from: /data2/g3/wrTest/RNG209/

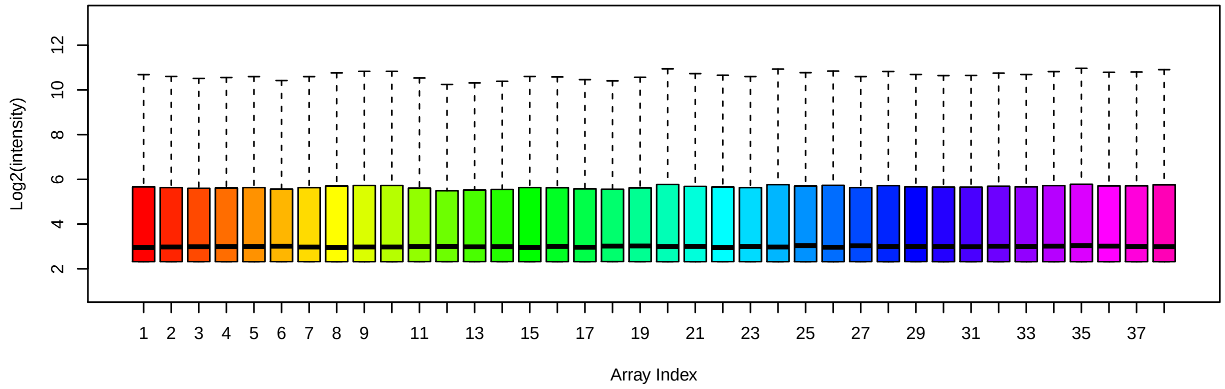

Distribution of GCRMA Processed Signal Intensity

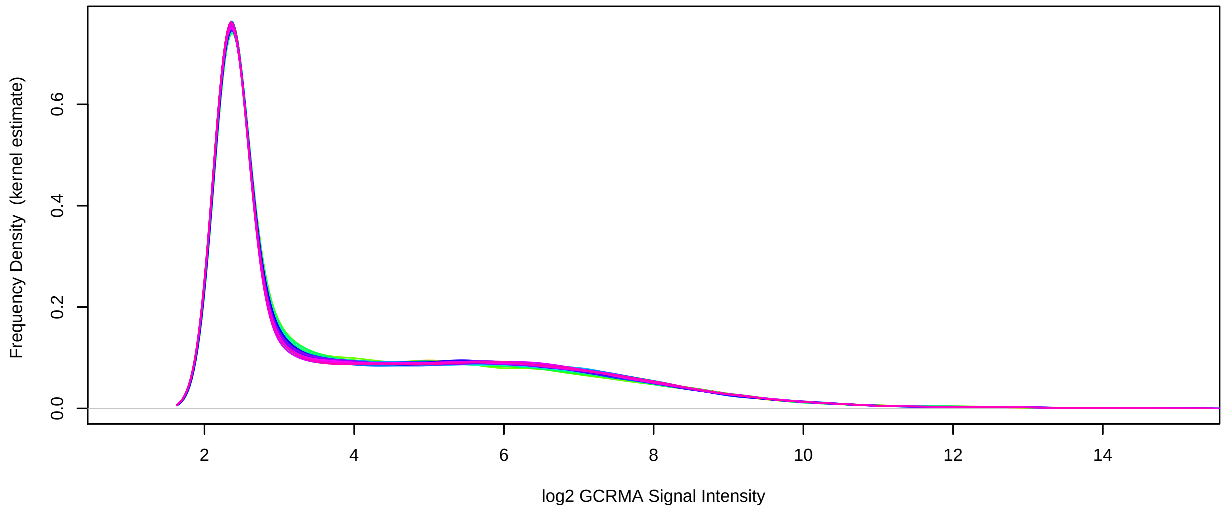

Similarity by GCRMA Summarized Samples (as Euclidean Distances)

using all probesets; n=54675, red indicates very high similarity

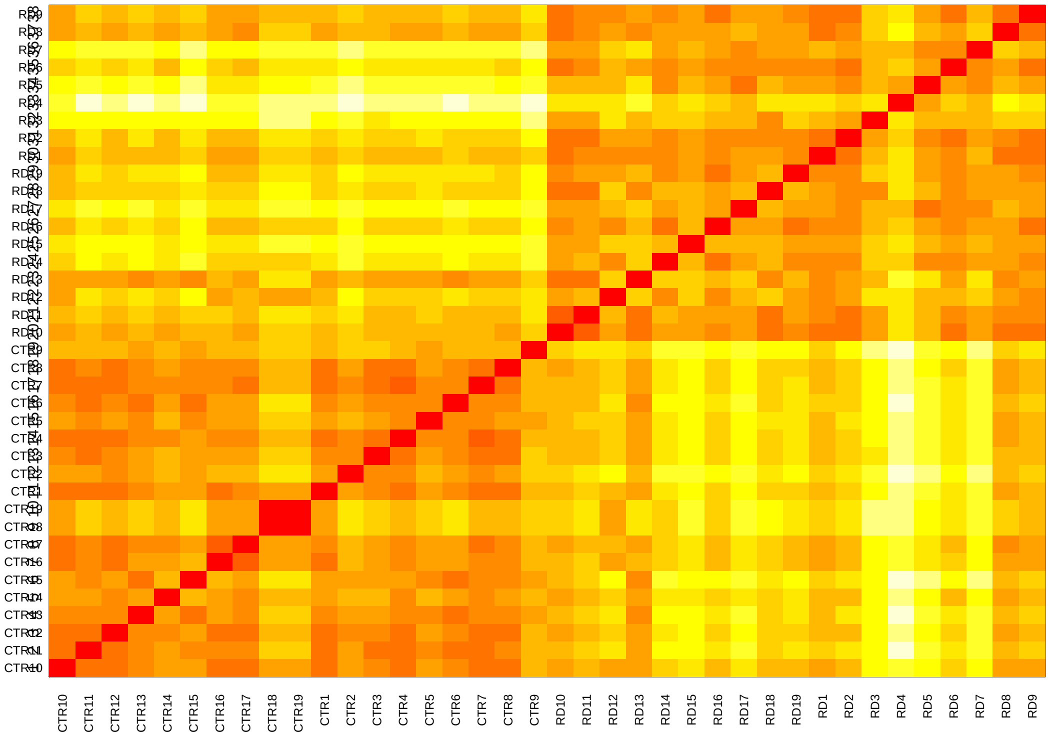

Dendrogram of Hierarchical Clustering of GCRMA data with Bootstrap p Values

using all probesets; n.bootstr=199; p values for AU (Approximately Unbiased) are shown in red, BP (Bootstrap Probability) in green

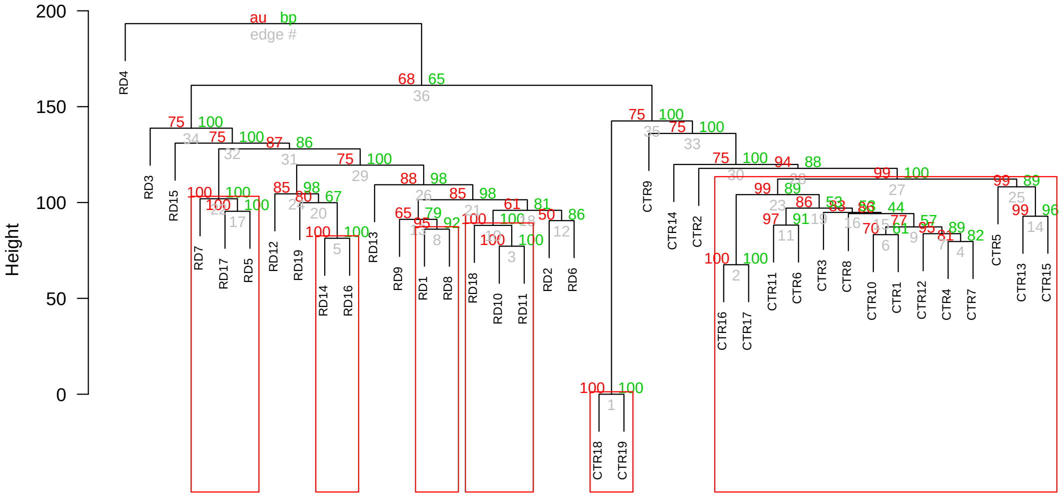

Clusters that 'seem to exist' with p>0.95 are highlighted by red boxes

Distance: euclidean
Cluster method: average

Principal Components Plot : 1st and 2nd Component

The numbers in the plot correspond to the 'array index' as listed on page 2 of this report

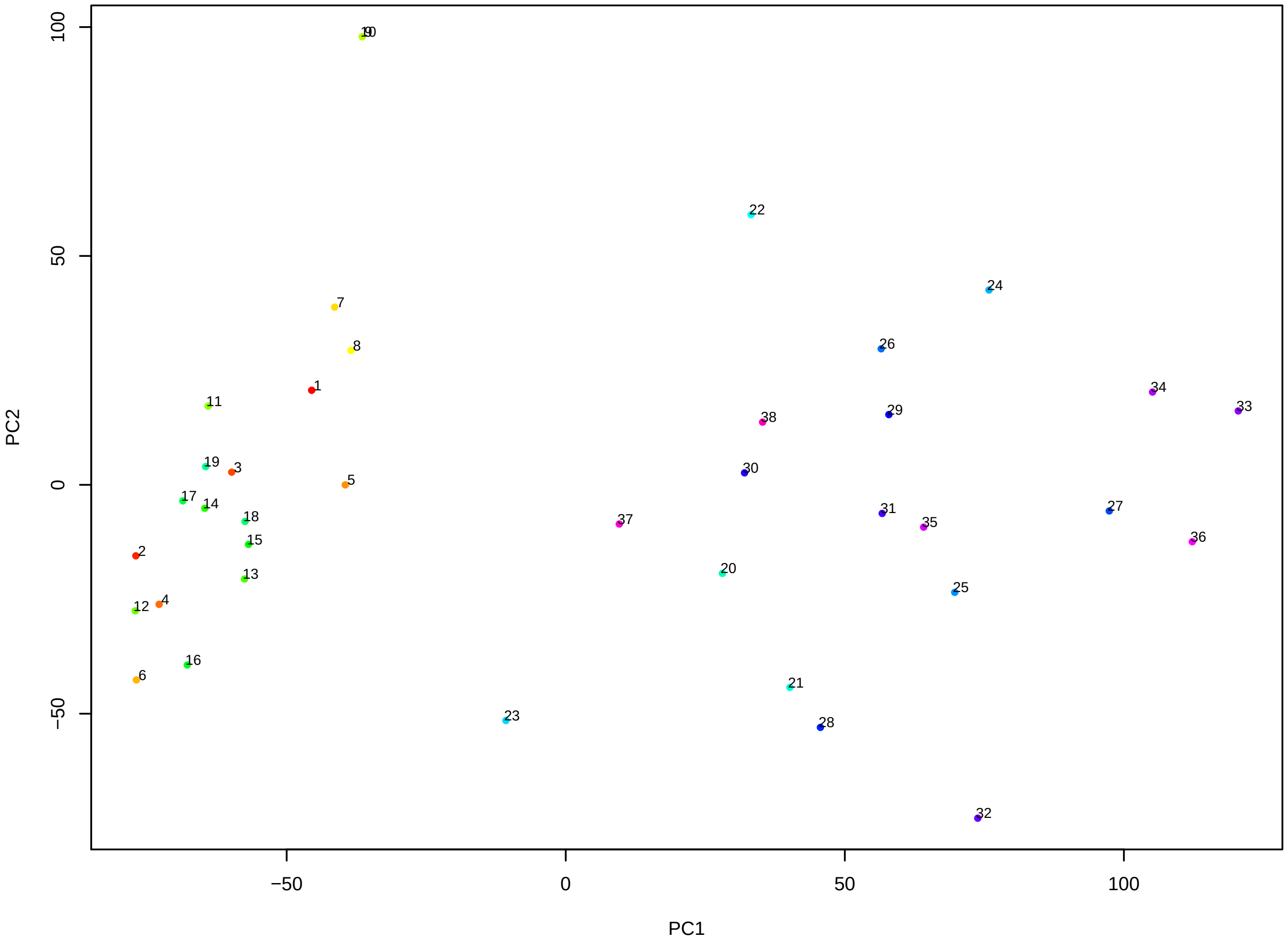

PCA : 2nd and 3rd Component

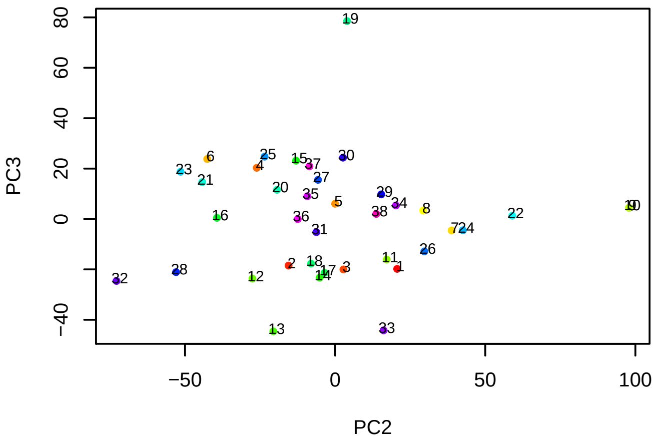

PCA : 1st and 3rd Component

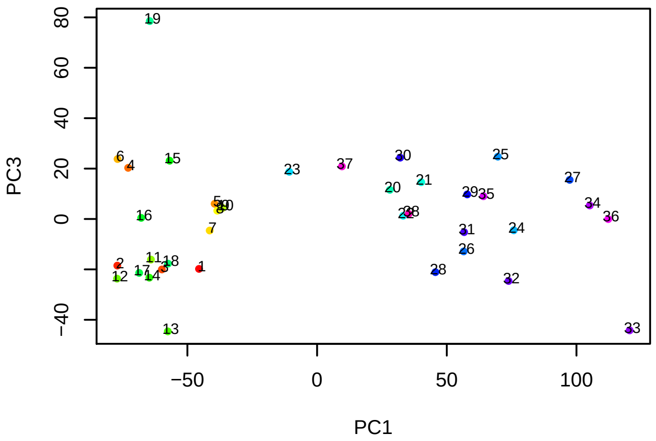

Screeplot on Variance Captured by the Principal Components

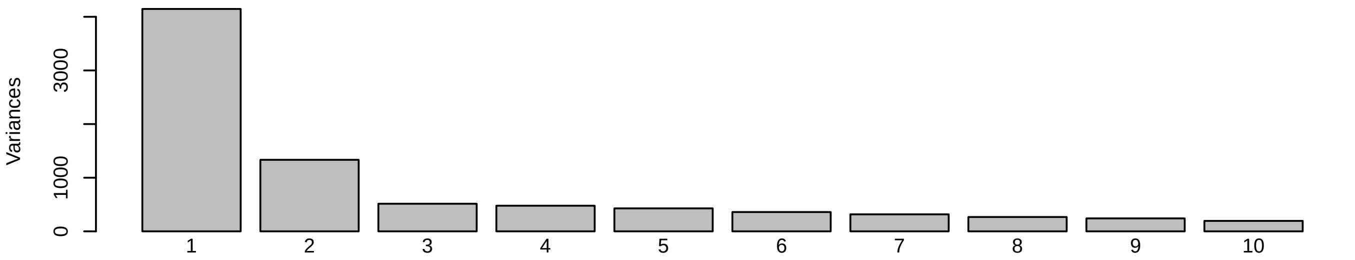

..total computing time : 335.3 min

Using R version 2.12.1 on a x86-64-unknown-linux-gnu system; dedicated R packages :

	Version No.
AnnotationDbi	1.12.0
Biobase	2.10.0
RColorBrewer	1.0-2
affy	1.28.0
affyPLM	1.26.0
affyQCReport	1.28.1
gcrma	2.22.0
genefilter	1.32.0
hgu133plus2cdf	2.7.0
hgu133plus2probe	2.7.0
lattice	0.19-17
preprocessCore	1.12.0
pvclust	1.2-1
simpleaffy	2.26.1
xtable	1.5-6

References :

- Raffelsberger W, Krause Y, Moulinier L, Kieffer D, Morand AL, Brino L and Poch O : RReportGenerator : Automatic reports from routine statistical analysis using R, Bioinformatics. 2008 Jan 15; 24(2): 276-278. and http://www-bio3d-igbmc.u-strasbg.fr/∼wraff
- R Development Core Team : R: A language and environment for statistical computing. R Foundation for Statistical Computing, Vienna, Austria. ISBN 3-900051-07-0, 2005 http://www.R-project.org.
- Leisch F : Sweave: Dynamic generation of statistical reports using literate data analysis. In: Härdle,W. and Rönz,B. (eds), Proceedings in Computational Statistics, Physica Verlag, Heidelberg, 2002 pp.575-580
- Gentleman RC, Carey VJ, Bates DM, Bolstad B, Dettling M, Dudoit S, Ellis B, Gautier L, Ge Y, Gentry J, Hornik K, Hothorn T, Huber W, Iacus S, Irizarry R, Leisch F, Li C, Maechler M, Rossini AJ, Sawitzki G, Smith C, Smyth G, Tierney L, Yang JY, Zhang J. : Bioconductor: open software development for computational biology and bioinformatics. Genome Biol., 2004, 5, R80.
- Bolstad BM, Irizarry RA, Astrand M, Speed TP : A comparison of normalization methods for high density oligonucleotide array data based on variance and bias. Bioinformatics. 2003 Jan 22;19(2):185-93.
- Irizarry RA, Bolstad BM, Collin F, Cope LM, Hobbs B, Speed TP : Summaries of Affymetrix GeneChip probe level data. Nucleic Acids Res. 2003 Feb 15;31(4):e15.
- Gautier L, Cope L, Bolstad BM, Irizarry RA : affy–analysis of Affymetrix GeneChip data at the probe level. Bioinformatics. 2004 Feb 12;20(3):307-15.
- Irizarry RA, Gautier L, Bolstad BM, Miller C, with contributions from Astrand M, Cope LM, Gentleman R, Gentry J, Halling C, Huber W, MacDonald J, Rubinstein B, Workman C and Zhang J : affy: Methods for Affymetrix Oligonucleotide Arrays. R package.
- Wilson CL, Miller CJ : Simpleaffy: a BioConductor package for Affymetrix Quality Control and data analysis. Bioinformatics. 2005 Sep 15;21(18):3683-5.
- Bolstad B : affyPLM: Methods for fitting probe-level models. R package. (2007) http://bmbolstad.com
- Parman C, Halling C, Gentleman R: affyQCReport: QC Report Generation for affyBatch objects. R package
- Reimers M, Weinstein JN : Quality assessment of microarrays: visualization of spatial artifacts and quantitation of regional biases. BMC Bioinformatics. 2005 Jul 1;6:166.
- Smyth GK : Limma: linear models for microarray data. In: 'Bioinformatics and Computational Biology Solutions using R and Bioconductor'. R. Gentleman, V. Carey, S. Dudoit, R. Irizarry, W. Huber (eds), Springer, New York, 2005, pages 397-420.
- Gentleman RC, Carey VJ, Huber W, Irizarry R, Dudoit S (editors): Bioinformatics and Computational Biology Solutions Using R and Bioconductor. Springer, 2005. ISBN: 0-387-25146-42005.
- http://www.affymetrix.com
